# Delineation of a Subgroup of the Genus *Paraburkholderia*, Including *P. terrae* DSM 17804^T^, *P. hospita* DSM 17164^T^, and Four Soil-Isolated Fungiphiles, Reveals Remarkable Genomic and Ecological Features—Proposal for the Definition of a *P. hospita* Species Cluster

**DOI:** 10.1093/gbe/evaa031

**Published:** 2020-02-18

**Authors:** Akbar Adjie Pratama, Diego Javier Jiménez, Qian Chen, Boyke Bunk, Cathrin Spröer, Jörg Overmann, Jan Dirk van Elsas

**Affiliations:** e1 Department of Microbial Ecology, Groningen Institute for Evolutionary Life Sciences, University of Groningen, The Netherlands; e2 Microbiomes and Bioenergy Research Group, Department of Biological Sciences, Universidad de los Andes, Bogotá, Colombia; e3 Leibniz Institute DSMZ-German Collection of Microorganisms and Cell Cultures, Braunschweig, Germany; e4 Department of Microbiology, Braunschweig University of Technology, Germany

**Keywords:** average nucleotide identity, comparative genomics, *Paraburkholderia hospita*, soil bacteria, species cluster

## Abstract

The fungal-interactive (fungiphilic) strains BS001, BS007, BS110, and BS437 have previously been preliminarily assigned to the species *Paraburkholderia terrae.* However, in the (novel) genus *Paraburkholderia*, an as-yet unresolved subgroup exists, that clusters around *Paraburkholderia hospita* (containing the species *P. terrae*, *P. hospita*, and *Paraburkholderia caribensis*). To shed light on the precise relationships across the respective type strains and the novel fungiphiles, we here compare their genomic and ecophysiological features. To reach this goal, the genomes of the three type strains, with sizes ranging from 9.0 to 11.5 Mb, were *de novo* sequenced and the high-quality genomes analyzed. Using whole-genome, ribosomal RNA and marker-gene-concatenate analyses, close relationships between *P. hospita* DSM 17164^T^ and *P. terrae* DSM 17804^T^, versus more remote relationships to *P. caribensis* DSM 13236^T^, were found. All four fungiphilic strains clustered closely to the two-species cluster. Analyses of average nucleotide identities (ANIm) and tetranucleotide frequencies (TETRA) confirmed the close relationships between *P. hospita* DSM 17164^T^ and *P. terrae* DSM 17804^T^ (ANIm = 95.42; TETRA = 0.99784), as compared with the similarities of each one of these strains to *P. caribensis* DSM 13236^T^. A species cluster was thus proposed. Furthermore, high similarities of the fungiphilic strains BS001, BS007, BS110, and BS437 with this cluster were found, indicating that these strains also make part of it, being closely linked to *P. hospita* DSM 17164^T^ (ANIm = 99%; TETRA = 0.99). We propose to coin this cluster the *P. hospita* species cluster (containing *P. hospita* DSM 17164^T^, *P. terrae* DSM 17804^T^, and strains BS001, BS007, BS110, and BS437), being clearly divergent from the closely related species *P. caribensis* (type strain DSM 13236^T^). Moreover, given their close relatedness to *P. hospita* DSM 17164^T^ within the cluster, we propose to rename the four fungiphilic strains as members of *P. hospita*. Analysis of migratory behavior along with fungal growth through soil revealed both *P. terrae* DSM 17804^T^ and *P. hospita* DSM 17164^T^ (next to the four fungiphilic strains) to be migration-proficient, whereas *P. caribensis* DSM 13236^T^ was a relatively poor migrator. Examination of predicted functions across the genomes of the seven investigated strains, next to several selected additional ones, revealed the common presence of features in the *P. hospita* cluster strains that are potentially important in interactions with soil fungi. Thus, genes encoding specific metabolic functions, biofilm formation (*pel*ABCDEFG, *pga*ABCD, alginate-related genes), motility/chemotaxis, type-4 pili, and diverse secretion systems were found.

## Introduction

A suite of studies has revealed members of the genus *Burkholderia* to be ubiquitous in soils (Salles et al. 2002) and plants (Stoyanova et al. 2007; Estrada-de los Santos et al. 2013; Sahl et al. 2015). However, key uncertainties have recently been identified with respect to the species boundaries inside and outside of this genus (Sawana et al. 2014; Beukes et al. 2017). Thus, Sawana et al. (2014) proposed a split of the genus *Burkholderia* into two novel genera, denoted *Burkholderia* and *Paraburkholderia*. Whereas the first genus comprises many human-associated and/or -pathogenic species, the second one encompasses mainly environmental (next to poorly characterized) species. A follow-up study by Estrada-de los Santos et al. (2016) revised this split and proposed two “transition” groups, denoted groups 1 and 2, in addition to two established clades (and a *Burkholderia* *andropogonis* group). Shortly following this, Beukes et al. (2017) showed evidence for the definition of four distinct genera: 1) *Paraburkholderia*, 2) *Caballeronia* (formerly transition group 2 of Estrada-de los Santos et al.), 3) *Burkholderia*, and 4) *Robbsia* (*andropogonis*). Very recently, Estrada-de los Santos et al. (2018) showed that two additional novel genera, that is, *Trinickia* and *Mycetohabitans*, also make part of *Burkholderia* “sensu lato.”

Within the genus *Paraburkholderia*, as defined by Sawana et al. (2014) and Beukes et al. (2017), a particularly interesting cluster of bacteria relevant for soil settings is formed by the species *Paraburkholderia* *terrae*, *Paraburkholderia* *hospita*, and *Paraburkholderia* *caribensis.* The type strains of these species, that is, *P. terrae* DSM 17804^T^, *P. hospita* DSM 17164^T^, and *P. caribensis* DSM 13236^T^, have been used as taxonomical and ecophysiological reference strains (Achouak et al. 1999; Goris et al. 2002; Yang et al. 2006). *Paraburkholderia* *terrae* DSM 17804^T^ was originally isolated from a forest soil in Daejeon, South Korea (Yang et al. 2006), whereas *P. hospita* DSM 17164^T^ came from an agricultural soil in Pittem, Belgium (Goris et al. 2002). The latter strain was isolated by virtue of its prominence as a key *in situ* recipient of the soil-introduced 2,4-dichlorophenoxyacetic acid (2,4-D) catabolic broad-host-range plasmids pJP4 and pEMT1 (Goris et al. 2002). Lastly, *P. caribensis* DSM 13236^T^ was first isolated from a vertisol soil in Martinique (French West Indies). Members of this species were found to produce high amounts of exopolysaccharides on carbon-rich media, indicating avid biofilm formation properties (Achouak et al. 1999).

In our initial studies on bacteria that can interact with soil fungi like *Laccaria proxima* and/or *Lyophyllum* sp. strain Karsten, particularly dominant bacteria were found to be able to migrate along fungal hyphae, form biofilms on these and grow on compounds present in fungal exudates (Warmink et al. 2011; Nazir et al. 2012). Many of the strains with these characteristics (notably BS001, BS007, BS110, and BS437) were initially assigned to the species *P. terrae* (Warmink et al. 2011; Nazir et al. 2012). All aforementioned strains were genome-sequenced in our lab, and the genome of one, BS001, was extensively examined (Haq et al. 2014). In this genome, several genes or operons were found to encode traits that enable establishment at, and interaction with, the hyphae of soil fungi. Thus, genes or operons encoding biofilm formation (Warmink et al. 2011), a type-three secretion (T3S) system (Yang et al. 2016), chemotaxis/flagellar movement, type-4 pili (T4P), and adherence traits were detected (Haq et al. 2014, 2016). Moreover, it was shown that comigration of strain BS001 with hyphae of *Lyophyllum* sp. strain Karsten growing through soil was critically dependent on the presence of flagella, with the T3S and T4P systems having minor effects (Yang et al. 2017). A newly found five-gene cluster that was presumably involved in energy generation from small molecules such as oxalate turned out to be highly upregulated when strain BS001 was placed in contact with *Lyophyllum* sp. strain Karsten (Haq et al. 2017), highlighting the importance of this gene cluster for fitness in the mycosphere.

Here, we hypothesize that an organismal clade related to the “classical” species *P. terrae* and *P. hospita* may have evolved in soil that shares genetic systems enabling its members to interact with soil fungi. At the time of writing of this manuscript, genomic data of the respective type strains of these two species, as well as of the comparator *P. caribensis* type strain, were unavailable. Thus, to enable genomic comparisons across these organisms, we determined their complete genome sequences using a combination of short- and long-read sequencing. Subsequently, we explored both the evolutionary relationships and ecological versatilities across all sequenced genomes and strains. The questions posed were: How related are the three type strains to one another and to the four aforedescribed fungiphilic *Paraburkholderia* strains? What are their unique versus common features? How may their evolutionary trajectory have shaped their modes of interaction with soil fungi? Can we find sets of genes or gene clusters that may be ascribed to such bacterial–fungal interactions?

## Materials and Methods

### Growth Conditions and Genomic DNA Extraction

*Paraburkholderia* *terrae* DSM 17804^T^, *P. hospita* DSM 17164^T^, and *P. caribensis* DSM 13236^T^ were cultured aerobically in Luria–Bertani (LB) medium, with shaking at 28 °C, 180 rpm (overnight). Genomic DNA was extracted using a modified protocol based on the UltraClean microbial DNA isolation kit (MOBio Laboratories Inc., Carlsbad, CA). The modification consisted of adding glass beads to the cultures in order to spur mechanical cell lysis. The extracted DNA was purified with the Wizard DNA cleanup system (Promega, Madison, WI), after which DNA quality and quantity were determined with a Nanodrop spectrometer (Thermo Scientific, Wilmington, DE). The qualities (degree of shearing) and quantities of the extracted DNAs were assessed using electrophoresis in 1% agarose gels.

### Metabolic Capacities of Strains and Interaction with Soil Fungi

Metabolic tests using BIOLOG GN2 (Biolog Inc., Hayward, CA) were performed for the strains according to the manufacturer’s protocol (Nazir et al. 2012). Briefly, early exponential-phase cultures were used as inocula for the Biolog test plates (150 µl per well). Each plate contained 96 microwells with one out of 95 different carbon sources in each and tetrazolium as an indicator of metabolic activity. Plates were incubated at 28 °C for up to 48 h to allow the observation of a purple color as an indicator of metabolic activity.

Interaction assays with soil fungi, in particular *Lyophyllum* sp. strain Karsten, were done according to Nazir et al. (2012). Briefly, single-strain migratory assays were done using Petri dishes with three compartments (Greiner Bio one, Frickenhausen, Germany), of which two were filled with presterilized (autoclaved) field soil (at 60% of water holding capacity, bulk density of ∼1.3 g/cm^3^ and 8 mm depth). The third compartment was filled with oat flake agar (OFA, 30 g/l oat flakes, 15 g/l agar) (Warmink et al. 2009) and served as a nutrient source for the fungus. Fresh (overnight) bacterial cultures were washed by centrifugation and resuspension in water, and then introduced evenly in a 3 mm-wide streak in the soil compartment directly adjacent to the front of the growing fungal hyphae, as well as in a similar system without fungal growth (negative control). The systems were incubated at 23 °C for 12–14 days of incubation. Following incubation, 100-mg soil portions at the hyphal fronts (“migration sites”) were punched out, shaken in liquid in Eppendorf tubes, and the resulting suspensions dilution-plated onto R2A plates. After incubation for up to 96 h at 28 °C, the plates were used for enumeration of the colony forming units (CFU). Strains with CFU numbers exceeding 10^7^ CFU per g soil at the migration site were considered to be good migrators, whereas strains with numbers below 10^5^ CFU per g represented poor migrators. In a second assay, *Lyophyllum* sp. strain Karsten was grown in propionate-containing minimal medium for two weeks at 28 °C. The culture was then harvested by centrifugation, after which the supernatant was filtered (0.45 µm pore size) and subsequently used as a medium to monitor the growth of selected *Paraburkholderia* strains (propionate cannot be utilized by the selected strains).

### Genome Sequencing and Assembly

Complete genome sequences of all three type strains (*P. terrae* DSM 17804^T^, *P. hospita* DSM 17164^T^, and *P. caribensis* DSM 13236^T^) were determined using a combination of two genomic libraries, of which one was prepared for sequencing with the PacBio *RSII* (Pacific Biosciences, Menlo Park, CA) platform. This SMRTbell template library was prepared and sequenced according to the instructions from Pacific Biosciences following the Procedure and Checklist “Greater than 10 kb Template Preparation and Sequencing.” Briefly, for preparation of 15 kb libraries, 5 µg genomic DNA was end-repaired and ligated overnight to hairpin adapters, applying components from the DNA/Polymerase Binding Kit P6 (Pacific Biosciences). Reactions were carried out according to the instructions of the manufacturer. BluePippin size selection to >4 kb was then performed (cf. Sage Science, Beverly, MA). Conditions for annealing of sequencing primers and binding of polymerase to purified SMRTbell template were assessed with the Calculator in RS Remote (Pacific Biosciences). SMRT sequencing was carried out on the PacBio *RSII* (Pacific Biosciences) taking one 240-min movie for one SMRT cell using the P6 Chemistry. Totals of around 712, 722, and 689 million bases were produced for *P. terrae* DSM 17804^T^, *P. hospita* DSM 17164^T^, and *P. caribensis* DSM 13236^T^, respectively. Paired-end short-read libraries for hybrid error correction were generated and sequenced on the Illumina HiSeq 2500 (Illumina, San Diego, CA) with 200 cycles resulting in ∼3.5 million paired-end reads per genome.

Long-read genome assemblies were generated using the “RS_HGAP_Assembly.3” protocol included in SMRTPortal version 2.3.0, applying default parameters, with the exception of *P. hospita* DSM 17164^T^, where the target genome size was set to 20 Mb. For *P. terrae* DSM 17804^T^ and *P. caribensis* DSM 13236^T^, four chromosomal contigs could be assembled, whereas the assembly of *P. hospita* DSM 17164^T^ led to five chromosomal contigs and the additional plasmid pEMT1. All assembled replicons were trimmed, circularized and adjusted to *dnaA* or their replication gene as the first gene. Total genome coverages of 52–61× were calculated within the long-read assembly process. Hybrid error correction was performed for each of the genomes by mapping of Illumina short-read data onto the draft circular genomes using BWA (Li and Durbin 2009) followed by automated variant calling using VarScan 2 (Koboldt et al. 2009) and GATK (McKenna et al. 2009) for consensus calling.

The genome sequences of *P. terrae* DSM 17804^T^, *P. hospita* DSM 17164^T^, and *P. caribensis* DSM 13236^T^ have been deposited at NCBI GenBank under accession numbers CP026111–CP026114, CP026105–CP026110, and CP026101–CP026104, respectively.

### Phylogenetic and Comparative Genome Analyses

To quickly check the quality of the sequencing and confirm previous (PCR-based) data, phylogenetic analyses were done based on the 16S rRNA genes of *P. terrae* DSM 17804^T^, *P. hospita* DSM 17164^T^, and *P. caribensis* DSM 13236^T^. Over 1,000 bp (including the V2–V6 regions) of the sequences were aligned using MUSCLE (Edgar 2004) and edited in accordance with Gblocks (Talavera et al. 2007). A maximum-likelihood tree was built with RAxML v.8.2.11 with nucleotide substitution model (GTRCAT), default algorithm setting (hill-climbing) and bootstrap value of 1,000 replicates (Stamatakis 2006). A second phylogenomics-based tree was constructed using the type (strain) genome server—TYGS (https://tygs.dsmz.de/; Meier-Kolthoff and Göker 2019). TYGS offers pairwise similarity calculation and a standard phylogenetic approach including multiple sequence alignment and analysis under the maximum-likelihood and maximum parsimony criteria. TYGS also allows the Genome BLAST Distance Phylogeny (GBDP) approach to rapidly infer trees with branch support values, which also enables calculation of dDDH values (Meier-Kolthoff and Göker 2019). Furthermore, trees were built by multilocus sequence analysis (MLSA) on the basis of the selected housekeeping genes *aroE, dnaE, groEL, gyrB, mutL, recA*, and *rpoB*. Each gene was aligned independently using MUSCLE (Edgar 2004) and edited in accordance with Gblocks (Talavera et al. 2007). The genes were then concatenated, aligned and edited as previously reported (Hug et al. 2016). The tree—based on maximum-likelihood—was built with RAxML v.8.2.11 using the amino acid substitution model PROTGAMMALG, hill-climbing algorithm, with bootstrap value of 1,000 replicates (Stamatakis 2006). Both phylogenetic trees were visualized using the “interactive tree of life” software (iTOL) v3 (Letunic and Bork 2016).

The MicroScope web platform hosted at Genoscope (MaGe) (Vallenet et al. 2017) was then used for genomic comparisons, and so the locus tags used by us are based on MaGe. The annotated high-quality genomes of *P. terrae* DSM 17804^T^, *P. hospita* DSM 17164^T^, and *P. caribensis* DSM 13236^T^ are publicly available in MaGe (http://www.genoscope.cns.fr/agc/microscope/home/index.php). Additionally, BlastN analyses were done for genomic comparisons and five-gene cluster searches for selected strains, that is, *P. hospita* (mHSR1 and LMG 20598), *P. caribensis* (strains MBA4, TJ182, Bcrs1W and MWAP64), and *P. terrae* (strain NBRC 100964).

Moreover, the gene sequence information of these type strains was further analyzed using TrEMBL, SwissProt, as well as comparisons to the PubMed and InterPro databases. To search for secreted proteins, SignalP was used. Finally, MicroScope also identified the relevant RNA genes (rRNA and tRNA).

Metabolic pathway analyses were done using two approaches. First, through the MicroScope platform, that is, using the microbial pathway/genome databases (PGDBs). Metabolic profile analysis was based on the computation of a “pathway completion” value, that is, the ratio between the number of reactions for pathway X in a given organism and the total number of reactions of pathway X defined in the database. Second, metabolic pathways were also inferred with the KEGG automatic annotation server (KAAS) (Moriya et al. 2007). Additionally, the secondary metabolite detection program AntiSMASH was used (Vallenet et al. 2017). The web server OrthoVenn (Wang et al. 2015) was used to compare the clusters of orthologous genes (COGs) between the genomes of *P. terrae* DSM 17804^T^, *P. hospita* DSM 17164^T^, *P. caribensis* DSM 13236^T^, next to BS007.

Average nucleotide identity values (ANIs) and tetranucleotide frequency correlation coefficients (TETRA) were obtained using JSpeciesWS (Richter et al. 2016). The measures of ANIs were done by the algorithms BLAST+ (ANIb) and MUMmer-Maximum Unique Matches (ANIm). Additionally, TETRA correlation search (TCS) analyses (shown as *Z*-values) were also done to provide a hit-list for insight into the relationships of the genomes with those of the reference genome database (Richter et al. 2016). Genes encoding carbohydrate-active enzymes (CAZymes; potentially involved in carbohydrate metabolism) were analyzed using dbCAN (Yin et al. 2012).

### Identification of Regions of Genomic Plasticity, Prophages, and CRISPR Spacers

Regions of genomic plasticity (RGPs) were predicted using MicroScope (at Genoscope; Vallenet et al. 2017). The platform employs “RGP finder” together with genomic island (GI) identifiers based on hidden Markov models (Waack et al. 2006) and AlienHunter-IVOM (Vernikos and Parkhill 2006). The GI identifier pipeline identified RGPs based on the criteria: 1) RGPs > 5 kb, 2) CDSs not belonging to conserved synteny groups between the compared organisms, and 3) regions with <50% of gene similarity with reference organisms were removed. RGPs in the three type strains were identified in comparison with the fungiphilic strains BS001, BS007, BS110, and BS437. Moreover, prophage sequences and CRISPR spacers were identified with PHAST (Zhou et al. 2011) and CRISPRFinder (Grissa et al. 2008), respectively. Strict criteria were used to identify complete prophages, as in Pratama et al. (2018).

## Results

### Rationale of This Study

A range of fungiphilic *Paraburkholderia* strains has been previously described with respect to their “eco-phenotype” (describing their capacities to interact with soil fungi in simulated soil settings). Many of these strains turned out to be loosely allocated in the species *P. terrae*, and the genome sequences of four such strains, that is, BS001, BS007, BS110, and BS437, have been described (Haq et al. 2014; Pratama et al. 2017). In order to enable a thorough analysis of the relationships of the fungiphilic strains with the three close relatives *P. terrae* DSM 17804^T^, *P. hospita* DSM 17164^T^, and *P. caribensis* DSM 13236^T^, determination of the phenotypic and genomic properties of the latter three type strains was a prerequisite. At the onset of this study, no such deeply sequenced genomes were available. Hence, we first assembled the relevant data sets regarding the genotypes (genome sequences), as well as the eco-phenotypes, of these three type strains. In a second stage, we compared the data of the former four fungiphilic strains to those that typify the type strains and determined their grouping.

### Summary of Phenotypic Traits of Type Strains *P. terrae* DSM 17804^T^, *P. hospita* DSM 17164^T^, and *P. caribensis* DSM 13236^T^

Microscopic studies of *P. terrae* DSM 17804^T^, *P. hospita* DSM 17164^T^, and *P. caribensis* DSM 13236^T^ cells confirmed that all three type strains had Gram-negative, rod-shaped and motile cells, as described (Achouak et al. 1999; Goris et al. 2002; Yang et al. 2006). On R2A plates, all three strains grew between 15 and 37 °C, and optimally at 28 °C. Based on BIOLOG GN2 assays, the three type strains, next to the fungiphilic strains BS001, BS007, BS110, and BS437, consistently utilized the following 53 of the 95 GN2 carbon sources tested: Tween-40, Tween-80, *N*-acetyl-d-glucosamine, adonitol, l-arabinose, d-arabitol, d-fructose, l-fucose, d-galactose, α-d-glucose, m-inositol, lactulose, d-mannitol, d-mannose, l-rhamnose, d-sorbitol, cis-aconitic acid, citric acid, formic acid, d-galactonic acid, lactose, d-galacturonic acid, d-gluconic acid, d-glucosaminic acid, d-glucuronic acid, α-hydroxy butyric acid, β-hydroxy butyric acid, p-hydroxy phenyl acetic acid, α-keto butyric acid, dl-lactic acid, quinic acid, d-saccharic acid, sebacic acid, succinic acid, bromo succinic acid, l-alaninamide, d-alanine, l-alanine, l-alanyl-glycine, l-asparagine, l-aspartic acid, l-glutamic acid, glycyl-l-glutamic acid, l-histidine, hydroxy-l-proline, l-ornithine, l-phenylalanine, l-proline, l-pyroglutamic acid, l-threonine, dl-carnithine, γ-amino butyric acid, urocanic acid, 2-aminoethanol, dl-α-glycerol phosphate, and glucose-6-phosphate (supplementary table 1*A*, Supplementary Material online).

Two additional compounds, that is, *N*-acetyl-d-galactosamine and manolic acid, were utilized by *P. terrae* DSM 17804^T^, *P. hospita* DSM 17164^T^, and the four fungiphiles, but not by *P. caribensis* DSM 13236^T^. In contrast, the compounds inosine, maltose, d-trehalose and methyl pyruvate could only be utilized by *P. caribensis* DSM 13236^T^.

These data indicate the metabolic versatility of these strains, in that most strains utilized a majority of the carbon sources of the BIOLOG system. A hierarchical cluster analysis based on the carbon compound utilization patterns showed a division in two clusters, one with *P. terrae* DSM 17804^T^, *P. hospita* DSM 17164^T^, and the four fungiphilic strains, and a distant one containing *P. caribensis* DSM 13236^T^ (supplementary fig. 1, Supplementary Material online).

Furthermore, *P. terrae* DSM 17804^T^ and *P. hospita* DSM 17164^T^ showed responses to compounds released by the fungus *Lyophyllum* sp. strain Karsten into propionate-supplemented mineral medium, which include glycerol, oxalate, citric acid, acetate and formate (>5-fold increased population sizes), whereas *P. caribensis* DSM 13236^T^ did not show such growth responses (table 1). This finding was consistent with data from experiments that showed *P. terrae* DSM 17804^T^ and *P. hospita* DSM 17164^T^ to be able to actively interact with *Lyophyllum* sp. strain Karsten in soil, in terms of showing single-strain migratory capabilities along with the soil-exploring fungal hyphae (table 1); this was similar to the behavior of fungiphilic strains BS001, BS007, and BS110 (here used as controls, previously observed as in Nazir et al. 2012). In contrast, *P. caribensis* DSM 13236^T^ was a poor migrator, with the connotation that its abundance at the inoculation site in soil increased in the presence of *Lyophyllum* sp. strain Karsten hyphae (see table 1).

**Table 1:** Fungal-Interactive Traits (Partially Modified From Nazir et al. [2012])

Strains	Survival at Inoculation Site^a^	Migration to Distal Site^a^	Response to Fungal Exudate (Propionate)^b^
*Paraburkholderia terrae* DSM 17804^T^	+	++	13
*Paraburkholderia hospita* DSM 17164^T^	+	+++	15
*Paraburkholderia caribensis* DSM 13236^T^	+	–	0
Strain BS001	+	+++	17
Strain BS007	+	++	16
Strain BS110	+	+++	23
Strain BS437	+	+	11

a Population sizes are given. +, log CFU/g 6.0–6.5; ++, log CFU/g 6.5–7.5; +++, log CFU/g 7.5–8.5.

b Approximate fold increase compared with *P. caribensis.*

On the basis of these collective data, we conclude that *P. terrae* DSM 17804^T^ and *P. hospita* DSM 17164^T^ had a very similar eco-phenotype, in particular with respect to their metabolic and fungal-responsive capacities, being akin to the four fungiphiles. This eco-phenotype was clearly divergent from that of *P. caribensis* DSM 13236^T^.

### Overall Analysis of the Genomes of *P. terrae* DSM 17804^T^, *P. hospita* DSM 17164^T^, and *P. caribensis* DSM 13236^T^

Deep sequencing and high-quality assembly of the three genomes revealed total genome sizes of 9.0–11.5 Mb. In detail, the size of the *P. terrae* DSM 17804^T^ genome was 10,062,489 bp (G + C content 61.79%), that of *P. hospita* DSM 17164^T^ 11,527,706 bp (G + C content 61.79%) and that of *P. caribensis* DSM 13236^T^ 9,032,490 bp (G + C content 62.58%). The genome of *P. terrae* DSM 17804^T^ was assembled into four, that of *P. hospita* DSM 17164^T^ into six (including the introduced plasmid pEMT1) and that of *P. caribensis* DSM 13236^T^ into four circular contigs, highlighting the number of separately replicating entities (supplementary table 2, Supplementary Material online). All assembled contigs were deposited as circular bacterial chromosomes, with the exception of the circular ∼100 kb contig in *P. hospita* strain DSM 17164^T^, which represents the full 2,4-D degradative plasmid pEMT1 that had been introduced earlier (Goris et al. 2002) (GenBank accession no. CP026110).

Totals of 8,752, 10,009, and 7,761 coding sequences (CDSs) were found on the genomes of *P. terrae* DSM 17804^T^, *P. hospita* DSM 17164^T^, and *P. caribensis* DSM 13236^T^, respectively. Moreover, the genome of *P. terrae* DSM 17804^T^ contained 18 ribosomal RNA (rRNA) and 60 tRNA encoding genes, that of *P. hospita* DSM 17164^T^ 21 and 67 and that of *P. caribensis* DSM 13236^T^ 18 and 61, respectively. A summary of these genome features is shown in supplementary table 1*A*, Supplementary Material online, the project information is in supplementary table 1*B*, Supplementary Material online and the genome statistics in supplementary table 1*C*, Supplementary Material online.

### Phylogenetic Analyses of the Set of Strains

**Fig. 1.**
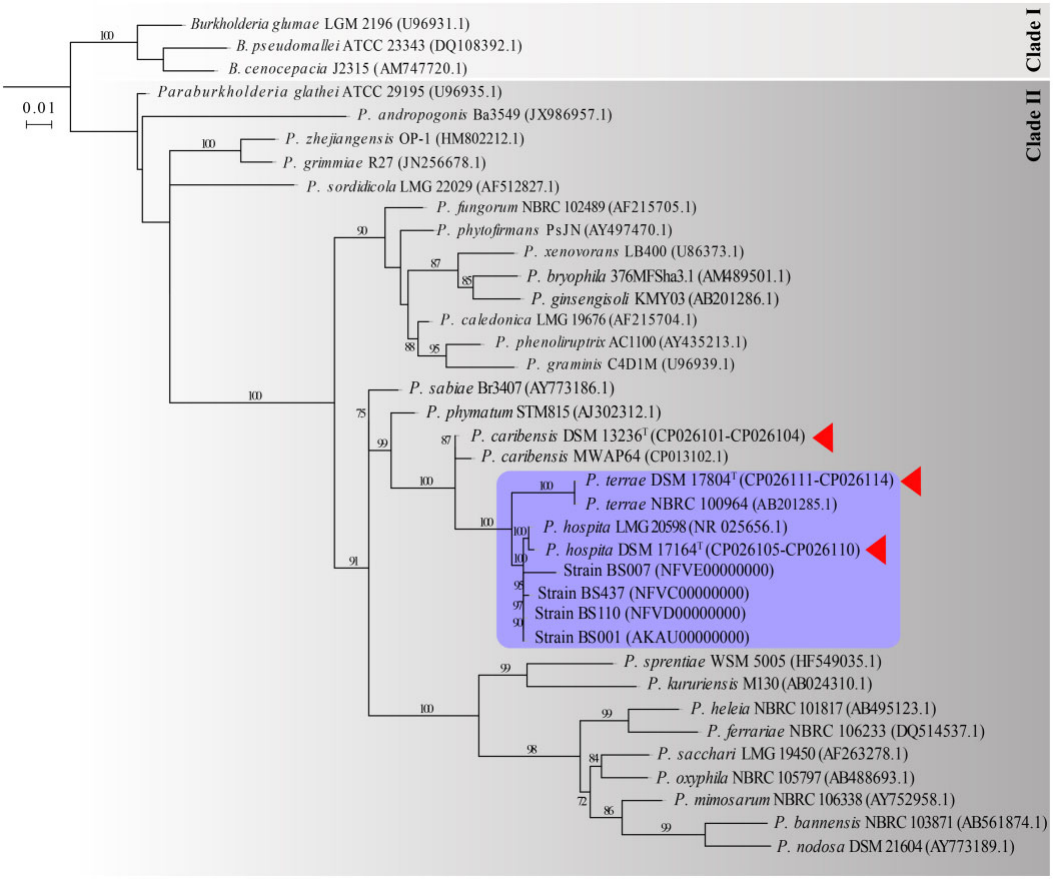
—Maximum-likelihood tree based on the V2–V6 (>1,000 bp) regions of the 16S rRNA gene. Accession numbers for the sequences used for each organism are provided in brackets after the organism’s name. The tree was constructed using RAxML (nucleotide substitution model GTRCAT), default algorithm settings (hill-climbing), with bootstrap value of 1,000 replicates. Bootstrap confidence values ≥ 70% are indicated. Purple box: proposed “species cluster.” Red triangles: type strains.

To verify and confirm the phylogenetic placement of the investigated strains on the basis of alignments of the 16S rRNA genes, a suite of relevant sequences across a selection of related *Paraburkholderia* species, including the close relative *Paraburkholderia* *phymatum*, was used. The analyses showed that *P. terrae* DSM 17804^T^, *P. hospita* DSM 17164^T^, and *P. caribensis* DSM 13236^T^ indeed clustered as a tight group within the genus *Paraburkholderia* (fig. 1). The former two species were most tightly knit (>98.67% reciprocal similarity), with the latter one being more distant (98.19% similarity with *P. terrae* DSM 17804^T^ and 98.47% with *P. hospita* DSM 17164^T^, see supplementary table 2, Supplementary Material online). The analysis confirmed *P. phymatum* to be a close relative to the whole species cluster. Moreover, *P. terrae* DSM 17804^T^ and *P. hospita* DSM 17164^T^ showed close relatedness to other (similarly named) *Paraburkholderia* strains, that is, *P. terrae* NBRC 100964 (AB201285.1) and *P. hospita* LMG 20598 (NR025656.1), respectively. Hence, this preliminary phylogenetic analysis produced a first glimpse of a possibly “tight” two-species cluster.

We then examined the precise placement of the four fungiphilic strains BS001 (NZ_AKAU00000000), BS007 (NFVE00000000), BS110 (NFVD00000000), and BS437 (NFVC00000000) versus this two-species cluster. Clearly, all four fungiphiles fell inside the cluster. Thus, using the strict criterion for species delineations, that is, up to 1–1.5% divergence of the 16S rRNA gene sequence, a tightly knit cluster of sequences, possibly indicating a “species cluster,” became discernible (fig. 1). This cluster showed a rather distant relatedness (96–98% similarity) to *P. caribensis* strains DSM13236^T^ and MWAP64 (CP013102.1), *P. phymatum* AJ302312.1 and *P. sabiae* AY773186.1. Interestingly, this is in agreement with data from Estrada-de los Santos et al. (2018).

**Fig. 2.**
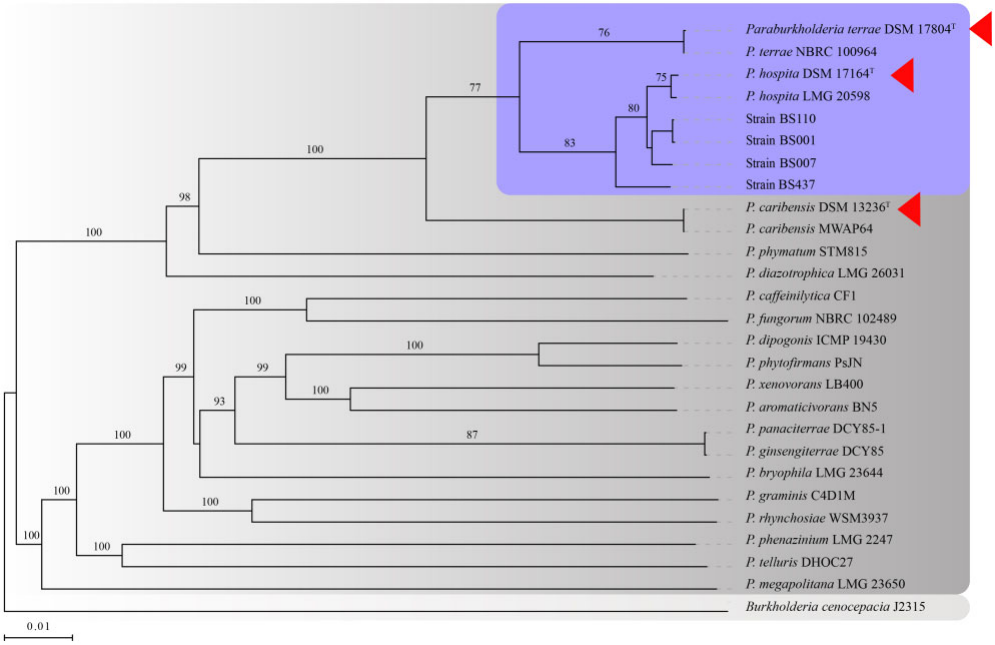
—Phylogenomics-based tree constructed using the type (strain) genome server—TYGS (https://tygs.dsmz.de/). Genome BLAST Distance Phylogeny (GBDP) distances were calculated from genome sequences. Branch lengths are scaled in terms of GBDP distance formula d_5_. Numbers above branches: GBDP pseudobootstrap support values from 100 replications. Bootstrap confidence values ≥ 70% are indicated. Purple box: proposed “species-cluster.” Red triangles: type strains.

**Fig. 3.**
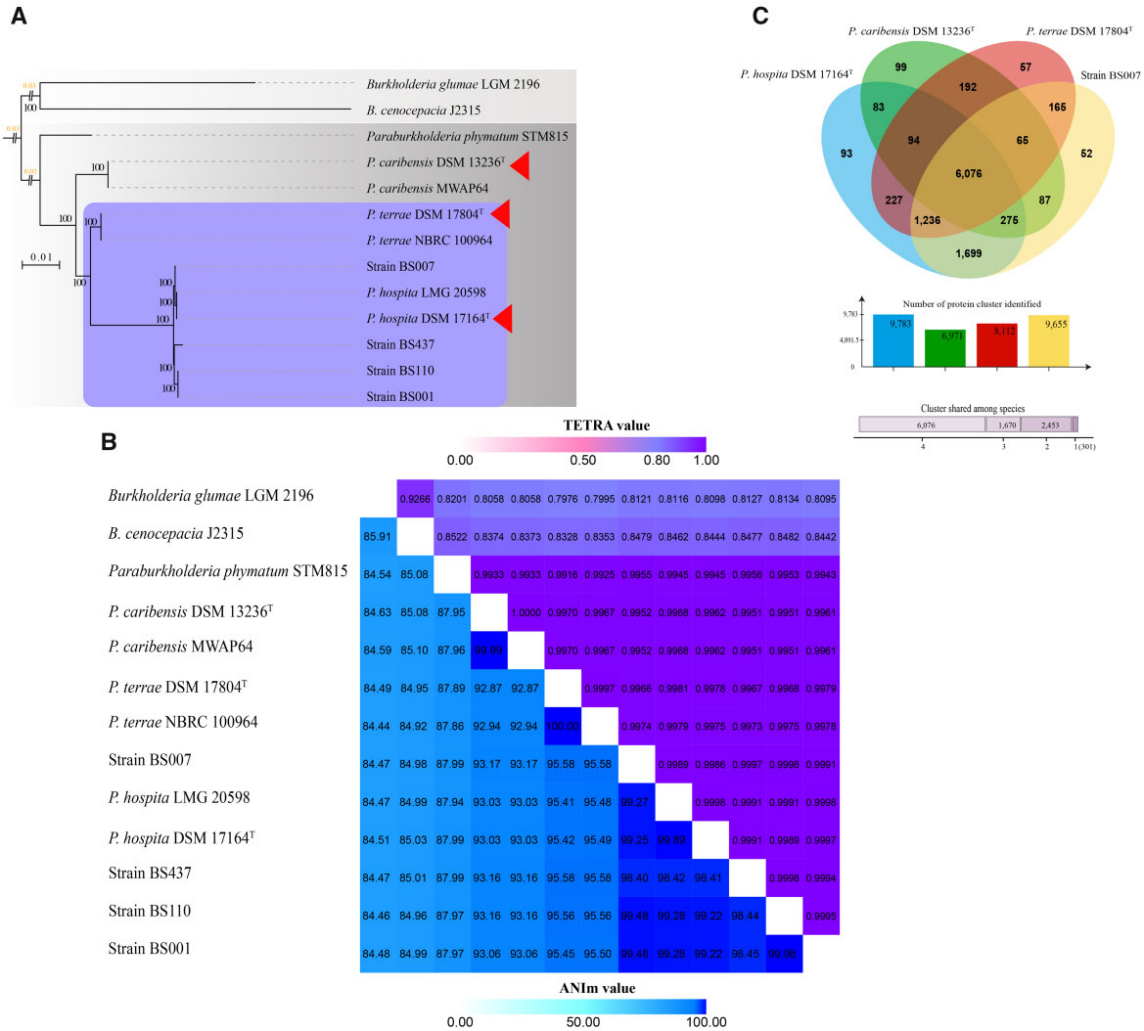
—(*A*) Maximum-likelihood phylogenetic tree based multilocus sequence analysis (MLSA) using seven concatenated core genes (*aroE, dnaE, groeL, gyrB, mutL, recA*, and *rpoB*). The tree was built with RAxML using amino acid substitution model PROTGAMMA, default matrix setting (Dayhoff) and algorithm (hill-climbing), with bootstrap value of 1,000 replicates. Bootstrap confidence values ≥ 70% are indicated. Purple box represents the proposed “species-cluster,” and red triangles indicate the type strains. (*B*) Heat maps of average nucleotide identity (ANIm) and tetranucleotide frequency (TETRA) analyses. The ANI (threshold 95–96%) and TETRA (>0.99) values were used for species circumscriptions (Richter and Rossello-Mora 2009). The ≥70% ANI coverage values were indicated. (*C*) Venn diagram of the orthologous clusters of proteins of *P. terrae* DSM 17804^T^, strain BS007, *P. hospita* DSM 17164^T^, and *P. caribensis* DSM 13236^T^. The number of protein clusters identified in each strain and shared protein clusters are indicated.

We then examined the whole-genome sequences across these strains to assess the validity of the initial analyses (fig. 2). This approach, as well as MLST analysis based on seven concatenated housekeeping genes (fig. 3*A*) yielded results that were fully consistent with the aforeshown clustering. Thus, the close relatedness of *P. terrae* DSM 17804^T^ and *P. hospita* DSM 17164^T^, as well as the divergence of *P. caribensis* DSM 13236^T^ from this two-species cluster, were confirmed. The two approaches (figs. 2 and 3*A*) also confirmed the tight relatedness between *P. hospita* DSM 17164^T^ and the four fungiphilic strains.

### ANI and TETRA Analyses

To explore the findings of similarity from the trees built on the basis of the 16S rRNA gene, the whole-genome sequences and the seven-gene concatenates, we assessed the evolutionary relationships between the three novel genomes under study (fig. 3*B*). As outgroups, we used genome sequences of *P. phymatum, Burkholderia cenocepacia*, and *Burkholderia* *glumae* (see fig. 3*B* and supplementary table 2, Supplementary Material online). The data indicated that *P. hospita* DSM 17164^T^, *P. terrae* DSM 17804^T^, and *P. caribensis* DSM 13236^T^ are only remotely related to either *P. phymatum* (ANIm: 87.89–87.99%, alignment: 60.36–61.91%) or the two *Burkholderia* strains (ANIm: 84–85%, alignment: 16–21%). Furthermore, the genomes of *P. hospita* DSM 17164^T^ and *P. terrae* DSM 17804^T^ were found to be highly similar across each other, with an ANIm of 95.42% (71.94% alignment). In contrast, the ANIm values between, on the one hand, *P. hospita* DSM 17164^T^ and *P. terrae* DSM 17804^T^ and, on the other hand, *P. caribensis* DSM 13236^T^ were only 93.03% (60.97% alignment) and 92.97% (69.61% alignment), respectively. These latter values are below those used for species delineations (threshold 95–96%) (Chun et al. 2018), and so the collective data confirm that *P. hospita* DSM 17164^T^ and *P. terrae* DSM 17804^T^ are 1) tightly related, at, or even within, species borders, and 2) divergent from *P. caribensis* DSM 13236^T^.

In addition, all four fungiphilic strains, that is, BS001, BS007, BS110, and BS437, fell inside this two-species cluster, being closer to *P. hospita* DSM 17164^T^ than to *P. terrae* DSM 17804^T^ (ANIm > 99%, 82–85% alignment, TETRA > 0.999). In detail, the genome of strain BS001 showed *Z*-score values of 0.99974 and 0.99789 against the genomes of *P. hospita* DSM 17164^T^ and *P. terrae* DSM 17804^T^, versus 0.99613 against that of *P. caribensis* DSM 13236^T^ (supplementary tables 3 and 4, Supplementary Material online).

### Clustering of Genes for Orthologous Proteins

Analyses of the COGs of the three type strains, next to the closely related strain BS007 (here selected for comparison) showed that the *P. terrae* and *P. hospita* type strains had high relatedness, with 227 COGs shared between them. Remarkably, 1,699 COGs were shared between *P. hospita* DSM 17164^T^ and strain BS007 (fig. 3*C*). In contrast, *P. hospita* DSM 17164^T^ and *P. caribensis* DSM 13236^T^ had only 83 COGs in common.

Furthermore, COG functional category analyses revealed the number of secondary metabolite biosynthesis functions (category Q) to be lower in *P. caribensis* DSM 13236^T^ (260 genes, 2.82%) than in both *P. hospita* DSM 17164^T^ (376, 3.12%) and *P. terrae* DSM 17804^T^ (344, 3.34%) (supplementary fig. 2, Supplementary Material online and supplementary table 5, Supplementary Material online).

### Insights into Potential Functions in *P. terrae* DSM 17804^T^, *P. hospita* DSM 17164^T^, and *P. caribensis* DSM 13236^T^ as Compared with Selected Fungiphilic Strains

#### Metabolic and Ecological Competence Traits

**Fig. 4.**
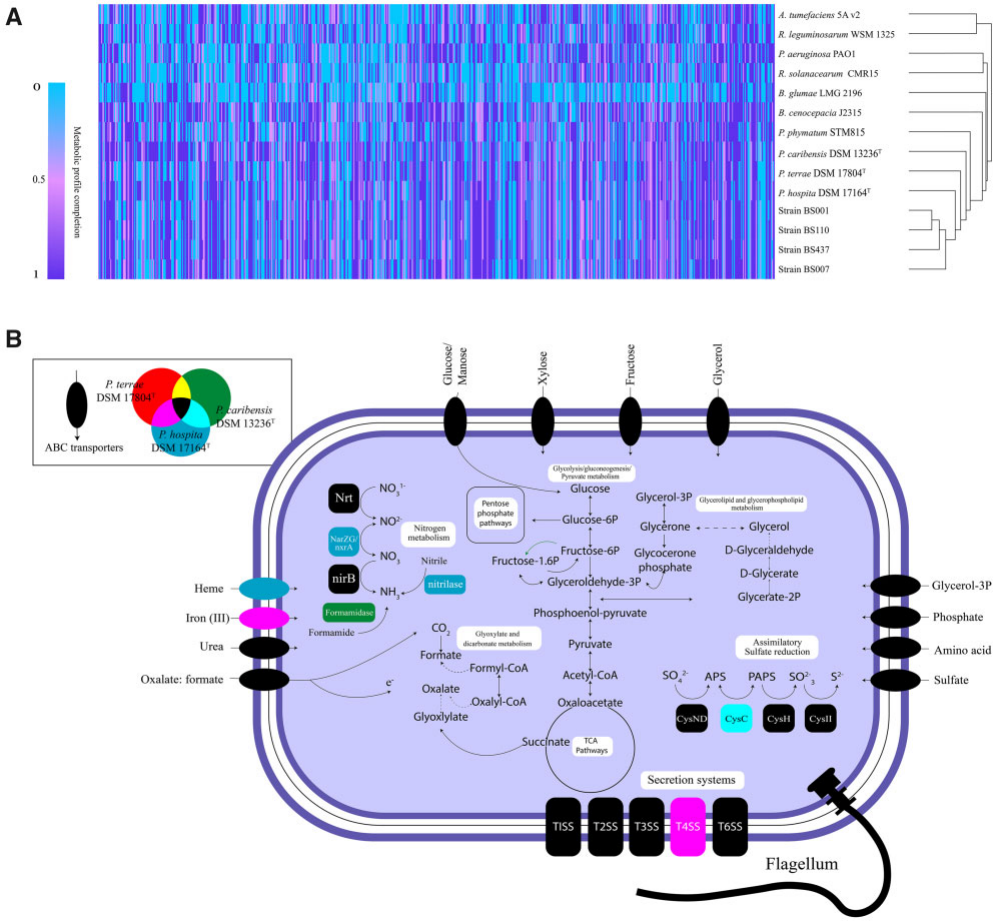
—(*A*) Hierarchical cluster analysis of metabolic profile completeness based on the Microscope platform. Computation of “pathway completeness” value: see Material and Methods. (*B*) Metabolic reconstruction of *Paraburkholderia terrae* DSM 17804^T^, *Paraburkholderia hospita* DSM 17164^T^ and *Paraburkholderia caribensis* DSM 13236^T^. The text in the white bubbles depicts names of pathways and metabolic processes. Pathways and corresponding enzymes were colored based on in which genome they were found. Moreover, incomplete pathways are indicated with dotted lines.

**Fig. 5.**
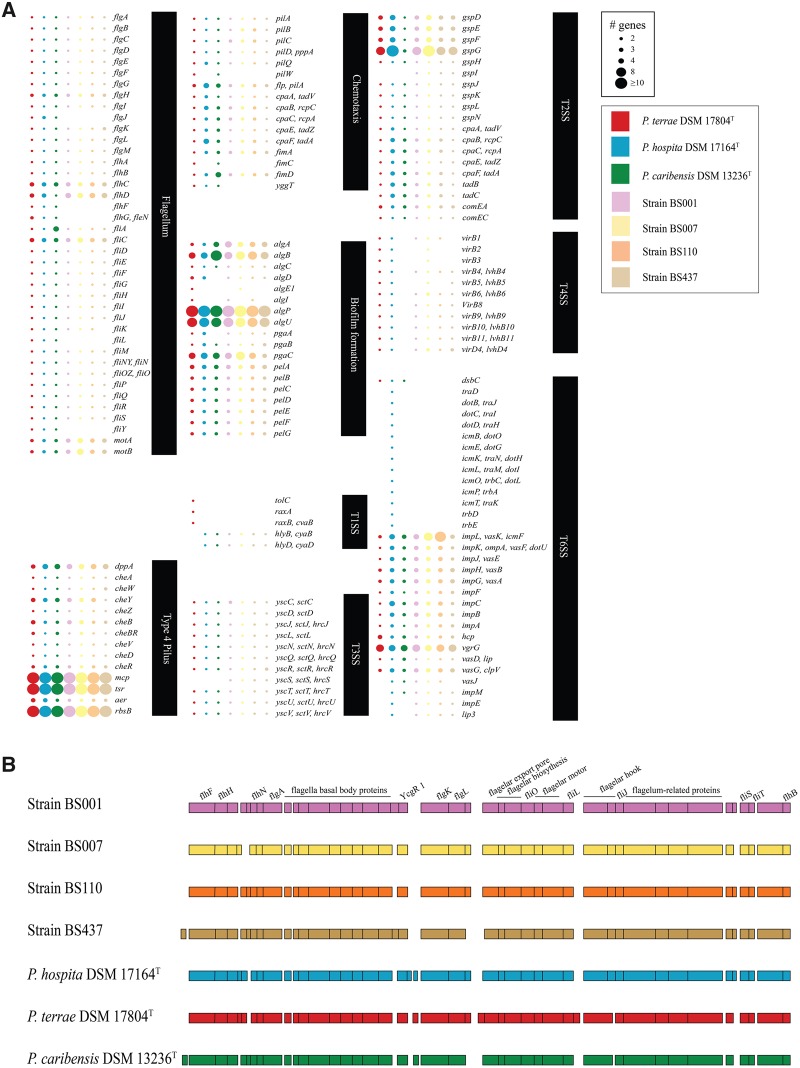
—(*A*) Gene number profile of selected genes in *Paraburkholderia terrae* DSM 17804^T^, *Paraburkholderia hospita* DSM 17164^T^, *Paraburkholderia caribensis* DSM 13236^T^ and four fungiphilic strains, i.e. BS001, BS007, BS110 and BS437. Color code based on the strain is indicated (*P. terrae* DSM 17804^T^: red; *P. hospita* DSM 17164^T^: blue; *P. caribensis* DSM 13236^T^: green; fungiphilic strains BS001: pink; BS007: yellow; BS110: orange; and BS437: brown). Number of genes is indicated by the dot size. (*B*) Synteny reconstruction of flagellar genes of *P. terrae* DSM 17804^T^, *P. hospita* DSM 17164^T^, and *P. caribensis* DSM 13236^T^. Color code based on the strain is indicated.

Expectedly, the genomes of the three type strains contained genes or operons for numerous diverse (primary and secondary) metabolic capacities (supplementary tables 6 and 7, Supplementary Material online), however without a clear distinction as to metabolic ranges. Across the genomes, sets of varying carbohydrate metabolism genes were found, indicating the presence of capacities to utilize simple (e.g., glucose, fructose) to complex (e.g., cellulose and hemicellulose) carbohydrates. Profile analyses showed that the genomes of the three type strains, in addition to those of all fungiphilic strains, possessed similar numbers and types of metabolic pathways, including the TCA cycle, glycolysis, the Entner–Doudoroff pathway, and gluconeogenesis (fig. 4*A* and *B*). Furthermore, they were predicted to be able to synthesize the essential amino acids histidine, isoleucine, leucine, lysine, methionine, phenylalanine, threonine, tryptophan and valine, the “conditional” amino acids arginine, cysteine, glutamine, glycine, proline and tyrosine, and the nonessential amino acids alanine, asparagine, glutamic acid, serine and selenocysteine. Evidence for the presence of several fermentation pathways was also found across the three genomes (fig. 4*B* and supplementary table 6, Supplementary Material online). Biofilm synthetic systems, that is, 1) pellicle formation (*Pel*—glucose-rich biofilm matrix exopolysaccharide), 2) poly-beta-1,6-*N*-acetyl-d-glucosamine (PGA—biofilm adhesin polysaccharide), and 3) alginate-related biofilm formation genes were found consistently (see fig. 5*A*). Likewise, sets of flagellar genes (fig. 5*B*) were found across the three type strains as well as the four fungiphilic strains. Diverse siderophore biosynthesis systems were also found (supplementary table 6, Supplementary Material online).

The genomes of *P. hospita* DSM 17164^T^ and *P. terrae* DSM 17804^T^, but not that of *P. caribensis* DSM 13236^T^, further revealed the presence of genes encoding the capacity to degrade 2-nitrobenzoate, anthranilate, alanine, 4-aminobutyrate and to synthesize trehalose. For more details, see supplementary table 6, Supplementary Material online. The capacity to degrade anthranilate has been linked to the early stage of biofilm formation in *Pseudomonas aeruginosa* (Costaglioli et al. 2012). This trait can also affect the structure of mushrooms at a later stage of biofilm formation (Kim et al. 2015).

Distinctive metabolic traits that were found to be uniquely encoded in the *P. caribensis* DSM 13236^T^ genome were: putrescine biosynthesis, oxidation of methanol to formaldehyde, fructose degradation, intra-aerobic nitrite reduction, methane sulfonate degradation, dissimilatory nitrate reduction, oxidation of GTP and dGTP, and hydrogen production.

There was a conspicuous absence of any gene encoding 6-phosphofructo-1-kinase (PFK-1; glycolysis pathway) from the genomes of *P. hospita* DSM 17164^T^ and *P. terrae* DSM 17804^T^ as well as from those of the fungiphilic strains BS001, BS007, BS110, and BS437. PFK-1 phosphorylates fructose 6-phosphate to fructose 1,6-bisphosphate. Conversely, this gene was found to be present in *P. caribensis* DSM 13236^T^ (fig. 4*B*). In all former strains, we assume this function to be taken over by the predicted kinase encoded by the *pfk*B gene.

#### Genes encoding CAZymes

**Fig. 6.**
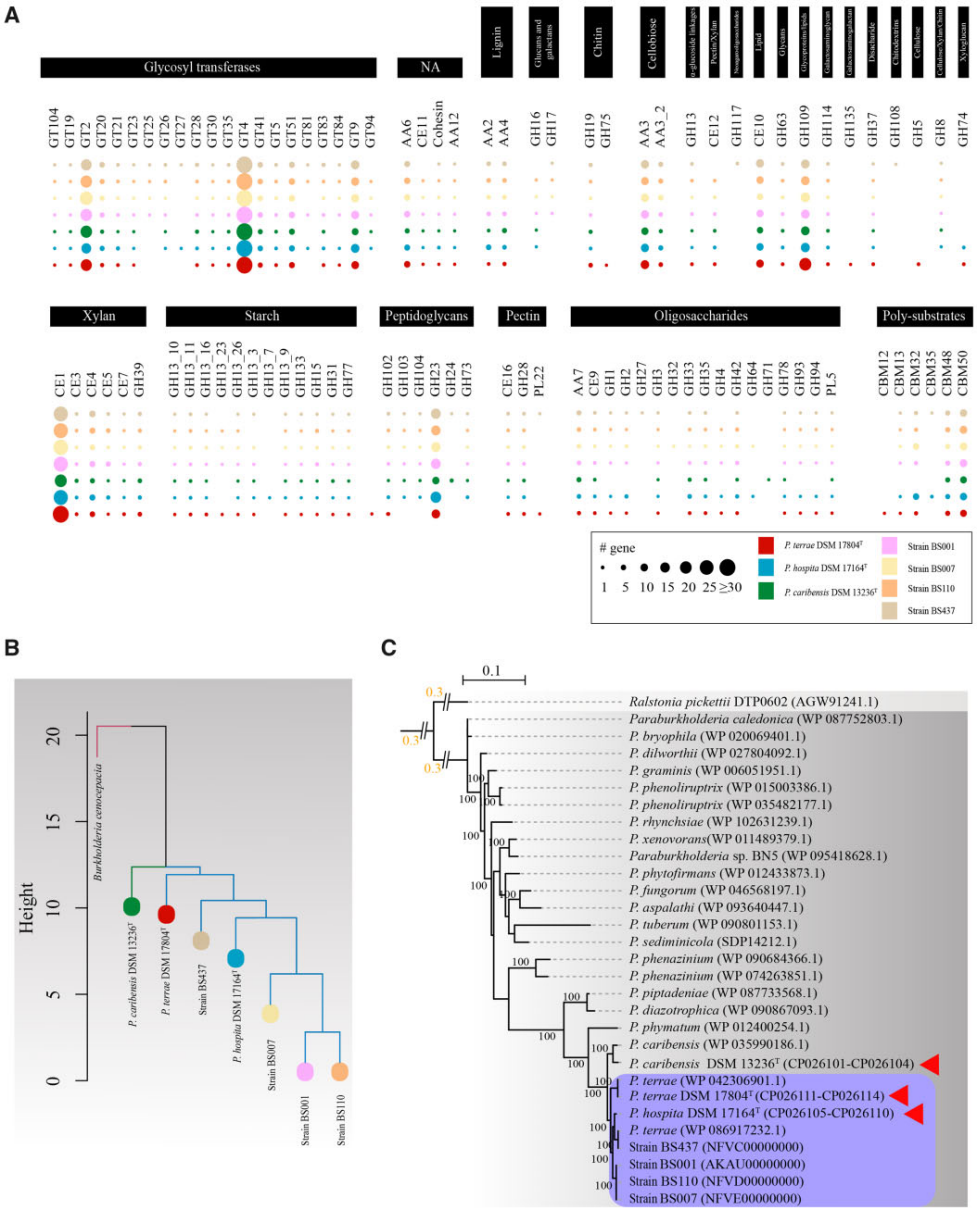
—(*A*) Gene number profile of predicted CAZYmes family proteins in *Paraburkholderia terrae* DSM 17804^T^, *Paraburkholderia hospita* DSM 17164^T^, and *Paraburkholderia caribensis* DSM 13236^T^, next to fungiphilic strains BS001, BS007, BS110, and BS437. Color code based on the strain is indicated. Number of genes is indicated by the dot size. (*B*) Hierarchical cluster analysis based on CAZyme gene number for *P. terrae* DSM 17804^T^, *P. hospita* DSM 17164^T^, *P. caribensis* DSM 13236^T^, strains BS001, BS007, BS110, and BS437. *B. cenocapacia* J2315 was used as an outgroup. (*C*) Phylogenetic analysis of GH3 type proteins across all strains, and the top hits of PSI-BLASTP. The tree was built with RAxML using amino acid substitution model PROTGAMMA, default matrix setting (Dayhoff) and hill-climbing algorithm, with bootstrap value of 1,000 replicates. Bootstrap confidence values ≥ 70% are indicated. Purple box represents the proposed “species-cluster,” and red triangles indicate the type strains.

The genomes of *P. terrae* DSM 17804^T^ and *P. hospita* DSM 17164^T^ contained, respectively, 302 and 308 genes encoding CAZymes, whereas those of fungiphilic strains BS001, BS007, BS110, and BS437 had 300, 310, 298, and 298 such genes (fig. 6*A*). In contrast, *P. caribensis* DSM 13236^T^ revealed a substantially lower number (253) of such genes. However, there was no major difference regarding the CAZyme or carbohydrate-binding module (CBM) profiles among all strains, including the type strains. Thus, all genomes revealed the presence of 31–39 genes for “auxiliary activity” (AA) family proteins, 14–26 for CBMs, 45–59 for carbohydrate esterases (CEs), 69–89 for glycosyl hydrolases (GHs), 92–109 for glycosyl transferases (GTs), and 1–2 for polysaccharide lyases (PLs). Overall, the most abundant genes predicted to encode CAZy family proteins were associated with: GT2, GT4, GT9 (family GTs), CE1 (carbohydrate esterases; involved in hydrolysis of xylan; acetyl xylan esterase [EC 3.1.1.72]), GH109 (glycosyl hydrolases, in particular glycoproteins; α-*N*-acetyl galactosaminidases involved in degradation of glycoproteins, EC 3.2.1.49), and AA3 (cellobiose dehydrogenase, EC 1.1.99.18) (fig. 6*A*).

Another suite of genes encoding CAZymes or CBMs was only found in *P. terrae* DSM 17804^T^ and *P. hospita* DSM 17164^T^ and not in *P. caribensis* DSM 13236^T^. The predicted proteins belonged to glycosyl hydrolase classes GH1, GH2, GH27, GH4, GH93, GH94, and GH74 and to CBM (carbohydrate binding moiety) classes CBM12, CBM13, and CBM32. In detail, moderate numbers (19–26) of genes encoding CBM family proteins were observed in both *P. hospita* DSM 17164^T^ and strain BS007, in particular those for CBM32 proteins (8). Interestingly, genes encoding CBM32 proteins were completely absent from the *P. caribensis* DSM 13236^T^ genome (fig. 6*A*).

A hierarchical cluster analysis based on the number of each of such genes per genome showed a close grouping of *P. terrae* DSM 17804^T^and *P. hospita* DSM 17164^T^ together with strains BS001, BS007, BS110, and BS437. In contrast, *P. caribensis* DSM 13236^T^ showed distant relatedness to this cluster (fig. 6*B*).

To address potential functional divergences, we then examined one example of a key GH3 family enzyme, that is, a glycoside hydrolase that removes single glycosyl residues and hydrolyzes bonds in cellulose, hemicellulose and starch (amylase) (Kusaoke et al. 2017). Genes for GH3 family proteins were found across all genomes examined here. The sequences of the identified genes were closely related, both between each other and with those of a suite of comparator *Paraburkholderia* strains. Based on the tree resulting from this analysis (see fig. 6*C*), the predicted GH3 protein in *P. caribensis* DSM 13236^T^ was strongly divergent from those of both *P. terrae* DSM 17804^T^ and *P. hospita* DSM 17164^T^.

#### Genes Encoding Membrane Transporters

The genomes of *P. terrae* DSM 17804^T^, *P. hospita* DSM 17164^T^, and *P. caribensis* DSM 13236^T^ contained genes predicted to encode a plethora of different membrane transporters. In particular, energy-dependent membrane transporter proteins, that is, ABC transporters, phosphotransferase systems (PTS), secondary membrane transporters (i.e., major facilitator superfamily; MFS), and solute carrier family (SLC) proteins were found (supplementary table 7, Supplementary Material online). We also found similar genes for aquaporins and small neutral solute transporters (supplementary table 8, Supplementary Material online). Finally, we found similar glycerol transporter and oxalate: formate antiporter (OFA) family transporters across all three type strains (see fig. 4*B* and supplementary table 8, Supplementary Material online).

Remarkably, a particular suite of genes encoding membrane transporters was only found in the *P. terrae* DSM 17804^T^ and *P. hospita* DSM 17164^T^ genomes, but not in that of *P. caribensis* DSM 13236^T^. This gene set included an iron (III) transporter system (*afuA/fbpA, afuB/fbpB*, *afuC/fbpC)* and the transmembrane electron carrier *torZ* (trimethylamine-N-oxide reductase; cytochrome c). Moreover, some genes for a few specific membrane transporters were unique per genome, that is, a predicted erythritol transporter in *P. terrae* DSM 17804^T^, arginine/ornithine and heme transporters in *P. hospita* DSM 17164^T^ and an organophosphate: P_i_ antiporter (OPA) family transporter in *P. caribensis* DSM 13236^T^ (supplementary table 8, Supplementary Material online).

#### Genes Encoding Motility Complexes

As expected, rather similar flagella-, T4P-, and chemotaxis-encoding gene complexes were found in the genomes of the three type strains, as well as in those of the four fungiphiles (fig. 5 and supplementary table 9, Supplementary Material online). The flagellar biosynthetic cluster was found to stretch over ∼45.3 kb, with high similarities across the whole region in *P. terrae* DSM 17804^T^, *P. hospita* DSM 17164^T^, all fungiphilic comparator strains, and *P. caribensis* DSM 13236^T^ (>90% similarity) (supplementary fig. 3, Supplementary Material online). Moreover, sets of chemotaxis genes, that is, *cheA, cheW, cheD, cheR, cheB, cheBR, cheY, cheZ*, and *cheV*, were found across these genomes. Genes encoding the T4P assembly proteins *pilABCDQW* were also found across the type strain genomes. However, a serine sensor receptor, that is, methyl-accepting chemotaxis protein I (*tsr*), could only be found in *P. hospita* DSM 17164^T^ (see fig. 5*A* and supplementary table 9, Supplementary Material online).

### Traits Predicted to Confer Associative Behavior with Soil Fungi

In this section, we analyze the genetic systems that are potentially related to mycosphere competence across the three type strains as compared with the fungal-interactive strain BS001. The mycosphere competence traits of strains BS007, BS110, and BS437 have been discussed earlier (Pratama et al. 2017).

#### Genes Encoding Secretion Systems

Genes/operons for type-1, -2, -3, and -6 secretion systems (T1SS, T2SS, T3SS, T6SS) were found across the genomes of all three type strains (see supplementary fig. 3, Supplementary Material online). In contrast, T4SS complexes, consisting of *VirB*1 through *VirB*11, in addition to *VirD4* (gene encoding the coupling protein key to conjugational DNA transfer) were only present in *P. terrae* DSM17804^T^ and *P. hospita* DSM17164^T^, but absent from the *P. caribensis* DSM 13236^T^ genome.

In detail, a complete T1SS system was found in *P. terrae* DSM 17804^T^ (i.e., OMP: *tolC*; MFP: *raxA*; and ABC: *raxB, cvaB*). In contrast, *P. hospita* DSM 17164^T^ and *P. caribensis* DSM 13236^T^ revealed genes for the ABC (*raxB, cvaB*), next to hemolysin D, proteins (fig. 5*A* and supplementary fig. 3, Supplementary Material online). With respect to the T2SSs, all three type strains contained genes for the nine canonical *gsp* genes (*gspD, gspE, gspF, gspG, gspH, gspJ, gspK, gspL*, and *gspN*). These T2SSs also contained tight adherence (*Tad*) export apparatuses, that is, *cpaA/tadV, cpaB/rcpC, cpaC/rcpA, cpaE/tadZ, cpaF/tadA, tadB*, and *tadC* (fig. 5*A*). With respect to the T3SSs, clusters containing the 19 canonical T3SS genes were identified across all three type strains. Ten of the 19 genes (*sctC, sctD, sctJ, sctL, sctN, sctQ, sctR, sctT, sctU*, and *sctV*) were highly syntenous, at ∼17–60% similarity (supplementary fig. 3, Supplementary Material online).

Finally, copies of the T6SS (consisting of the core component of the *imp/vas* secretion system) were found across the three type strains (supplementary fig. 3, Supplementary Material online). With respect to the *imp* system, one copy was found in *P. terrae* DSM 17804^T^, three in *P. hospita* DSM 17164^T^ and two in *P. caribensis* DSM 13236^T^. High synteny was found among the copies (denoted as cluster) in *P. terrae* DSM 17804^T^, *P. hospita* DSM 17164^T^ (cluster 3) and fungiphilic strain BS001 (cluster 1), indicating high relatedness. In addition, the T6SS cluster 1 of *P. hospita* DSM 17164^T^ was highly syntenous to that of *P. caribensis* DSM 13236^T^, as well as strain BS001 cluster 3 (Haq et al. 2014). Also, high synteny was observed between the T6SS clusters 2 in *P. hospita* DSM 17164^T^ and in strain BS001 (supplementary fig. 3, Supplementary Material online).

#### Genes Encoding Glycerol and Oxalate Metabolism, and Five-Gene Cluster

The analyses of the genomes of the three type strains did not detect the glycerol uptake gene *gup* that was previously discovered in the strain BS001 genome (Haq et al. 2014). However, glycerol uptake/transporter genes *glpV, glpP, glpO*, and *glpS* and putative *sn*-glycerol 3-phosphate transporter genes (*ugpB, ugpA, ugpE*, and *ugpC*) were found across the genomes of all three type strains (see supplementary fig. 4, Supplementary Material online).

We further found sets of genes predicted to encode proteins involved in oxalate (and formate) oxidation in all three type strains (supplementary fig. 4*B*, Supplementary Material online). A high similarity of one gene, that is, oxalyl-CoA decarboxylase, was found in the genomes of *P. terrae* DSM 17804^T^ (99%) and *P. hospita* DSM 17164^T^ (100%), next to that of strain BS001, suggesting these organisms have similar responsive behavior to oxalate. The relatedness of this gene sequence to the copy in *P. caribensis* DSM 13236^T^ was lower (98%).

**Fig. 7.**
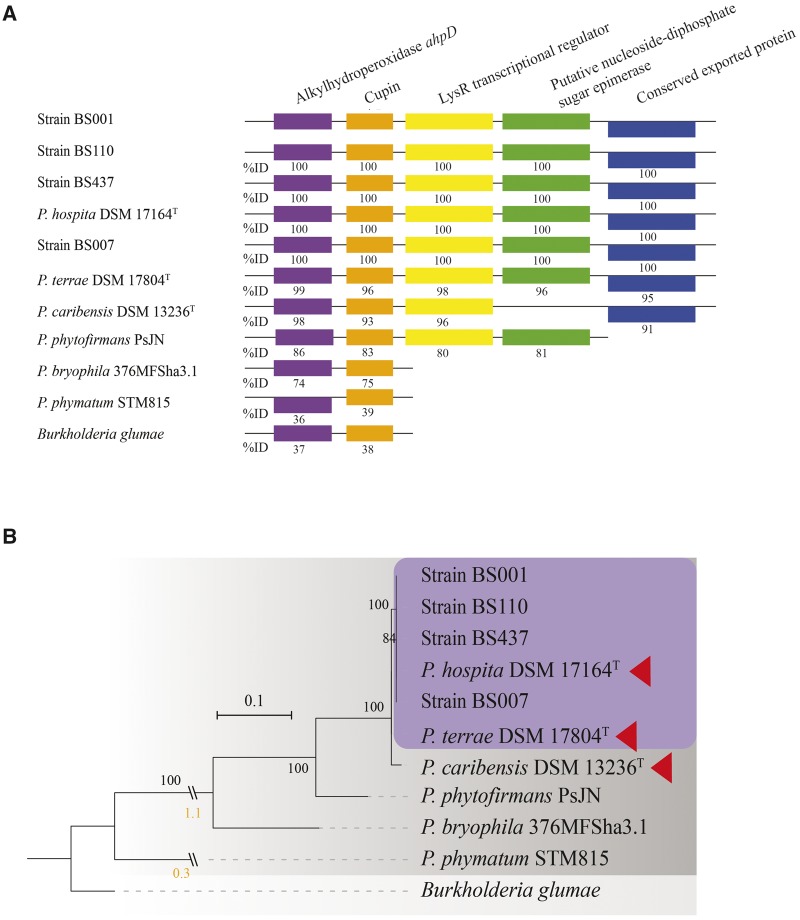
—(*A*) Comparison of the five-gene cluster among the strains. Comparison percentage using *Paraburkholderia terrae* strain BS001 as a reference and based on the Microscope genoscope platform. (*B*) Phylogenetic analysis of alkyl hydroperoxidase *AhpD* across *Paraburkholderia* species, including type strains and mycosphere-derived strains. *Burkholderia glumae* was used as an outgroup. The tree was built with RAxML using the amino acid substitution model PROTGAMMA, default matrix setting (Dayhoff) and hill-climbing algorithm. Bootstrap value 1,000 replicates. Bootstrap confidence values ≥ 70% are indicated. Purple box represents the proposed “species-cluster,” and red triangles indicate the type strains.

Remarkably, full copies of the five-gene cluster first found in strain BS001 and hypothesized by Haq et al. (2017) to be involved in the generation of energy from small carbonaceous molecules released by soil fungi (e.g., oxalate), as well as removal of concomitant oxidative toxicity, were found in both *P. terrae* DSM 17804^T^ and *P. hospita* DSM 17164^T^. Moreover, the genomes of strains BS007, BS110, and BS437 also contained the full gene cluster (fig. 7*A* and *B*). Although the cluster was also found in the *P. caribensis* DSM 13236^T^ genome, one gene (IV, encoding a putative nucleoside-diphosphate sugar epimerase) was lacking (fig. 7*A*). The relatedness of the five-gene cluster was close between *P. terrae* DSM 17804^T^ and *P. hospita* DSM 17164^T^ and more distant when compared with *P. caribensis* DSM 13236^T^, as illustrated in the phylogenetic analysis of the gene for alkyl hydroperoxide *ahpD* across the *Paraburkholderia* species (fig. 7*B*).

### RGPs, Prophage-Related Sequences and CRISPR-Cas Arrays

The genomes of all three type strains were found to contain multiple sets of RGPs. In detail, *P. terrae* DSM 17804^T^ and *P. hospita* DSM 17164^T^ had 97 and 99 RGPs, whereas *P. caribensis* DSM 13236^T^ had only 76 (supplementary table 9, Supplementary Material online). The total sizes of the RGPs were 3,009,744 (29.9% of the genome), 4,401,854 (38.2%) and 2,133,117 bp (23.6%), respectively.

The largest RGP (RGP72; 308 CDS) in *P. terrae* DSM 17804^T^ was 283,846 bp in size, that in *P. hospita* DSM 17164^T^ 1,365,074 bp (spanning one of the six contigs, and so resembling a megaplasmid—RGP98; 1,480 CDS) and that in *P. caribensis* DSM 13236^T^ 267,272 bp (RGP75; 302 CDS). RGPs containing complete T4SSs, thus indicating integrated plasmids (like RGP97 in BS001; Haq et al. 2014) were found in *P. hospita* DSM 17164^T^ (RGP99: 419,946 bp) as well as *P. terrae* DSM 17804^T^ (RGP97: 157,478 bp), but not in *P. caribensis* DSM 13236^T^.

Furthermore, genes encoding both predicted transposases and integrases were amply found in the *P. terrae* DSM 17804^T^ (42 and 11, respectively), *P. hospita* DSM 17164^T^ (154/44), and *P. caribensis* DSM 13236^T^ genomes (36/8). In all three type strains, these were located inside RGPs (supplementary table 9, Supplementary Material online).

Analyses of putative prophage (PP) regions across the type strains using PHAST (Zhou et al. 2011) identified one (25 kb) in *P. terrae* DSM 17804^T^, two in *P. hospita* DSM 17164^T^ (total size 40.7 kb), and two (total size 89.9 kb) in *P. caribensis* DSM 13236^T^ (supplementary table 10, Supplementary Material online). The genes in the two PP regions in *P. hospita* DSM 17164^T^ were assigned as mobile genetic elements (MGEs) related genes (no specific hits in the database), with mainly genes encoding hypothetical proteins and integrases, without phage structural genes (e.g., capsid, tail, terminase) being identified. Furthermore, the single 25 kb PP region in *P. terrae* DSM 17804^T^ probably represents an intact prophage (denoted as ϕPt17804), similarly to the two sequences in *P. caribensis* DSM 13236^T^ (yielding ϕPcari1DS and ϕPcari2DS).

Finally, we analyzed the three type strain genomes for the presence of CRISPR-Cas spacer sequences. Using CRISPR-Finder, we found 13, 14 and 9 CRISPR spacer sequences in *P. terrae* DSM 17804^T^, *P. hospita* DSM 17164^T^, and *P. caribensis* DSM 13236^T^, respectively. These spacer sequences matched sequences in a large variety of phage families, mostly identified as Myoviridae. Detailed analyses of the evolutionary history of prophages in the genomes of *Paraburkholderia* species are discussed in Pratama et al. (2018).

### Genome Comparison with Other Strains in the Species *P. hospita*, *P. caribensis*, and *P. terrae*

In the course of this work, several new genome sequences of *P. hospita, P. terrae*, and *P. caribensis* became available, next to sequences of some newly identified *Paraburkholderia* spp. Hence, we extended our comparative whole-genome analyses to these strains, in a separate analysis. The additional analyses included *P. caribensis* strains MBA4, TJ182, Bcrs1W, and MWAP64, *P. terrae* NBRC 100964 and *P. hospita* strains mHSR1 and LMG 20598 (supplementary fig. 5, Supplementary Material online). The data provide strong evidence for the contention that *P. hospita* and *P. terrae* are indeed tightly linked within one species cluster, which includes the four fungiphilic strains BS001, BS110, BS007, and BS437. Moreover, all *P. caribensis* strains clustered clearly as a sister group, separate from the former species cluster. All other strains used in the comparison clustered remote from these two sister groups. Moreover, genome size comparisons revealed the two-species cluster strains (especially *P. hospita)* to have large genomes (up to over 11 Mb), exceeding those of the *P. caribensis* strains (average ∼8 Mb), next to the other comparator genomes (i.e., of *P. phymatum*, *P. azotifigens*, *P. piptademiae*, and *P. diazotrophica;* average ∼9 Mb).

A search, across the additional genomes, for traits that might be involved in associative behavior with soil fungi revealed the presence of many such traits, that is, secretion systems, flagella, chemotaxis, glycerol-oxalate related and biofilm formation genes across these. The exception was the aforementioned five-gene cluster, as outlined below. Thus, all genomes did contain genes encoding secretion systems of the T2SS, T3SS, T4SS, and T6SS classes, with just *P. hospita* LMG 20598 and *P. caribensis* MWAP64 having a T1SS. Furthermore, across the genomes, we also found biofilm synthesis systems of the *Pel* and PGA classes. Complete sets of flagellar and chemotaxis genes (i.e., *cheD*, *cheR, cheB, cheA, cheW, cheV, cheZ*, and *cheY)* and genes for glycerol transporters and OFAs were also found across these strains. Specifically, genes *glpV, glpP, glpQ, glpS* and *glpT*, and *ugpB, ugpA, ugpE* and *ugpC* were found. Very interestingly, the complete five-gene cluster was found only in the additional *P. terrae* and *P. hospita* species, with incomplete versions of this cluster being detected in *P. caribensis* strains Bcrs1W and TJ182. This gene cluster was completely absent from *P. caribensis* strains MBA4 and MWAP64 (supplementary fig. 5, Supplementary Material online).

## Discussion

### Phenotypic Traits—Interactivity with Soil Fungi

In this study, we examined the genomic and metabolic/fungal-interactivity traits across the type strains of *P.* *terrae*, *P. hospita*, and *P. caribensis*, next to selected other (fungiphilic) strains of this genus, in order to delineate species boundaries and analyze fungal interactivity as a potentially common complex trait. All strains had a soil origin, and, to date, it has remained unexplored to what extent they would cluster together, or are divergent, with respect to their genomic and ecophysiological features. Moreover, we here report and analyze the deeply sequenced, assembled and annotated high-quality genomes of the three key type strains.

First, the clear evidence found for the tenet that *P. hospita* DSM 17164^T^ and *P. terrae* DSM 17804^T^ (but not *P. caribensis* DSM 13236^T^) can migrate through soil along with the hyphae of *Lyophyllum* sp. strain Karsten, placed these two strains in the category of “single-strain migratory” fungal-interactive strains, much like the comparator strains BS001, BS007, BS110, and (although weaker) BS437, that had previously been loosely assigned to the species *P. terrae* (table 1). Additionally, the metabolic complement of the three type strains, although fairly comparable, allowed a grouping in two clusters, one including *P. caribensis* and the other one all other organisms. It should be noted that *P. hospita* DSM 17164^T^ and *P. terrae* DSM 17804^T^ were able to utilize the compounds glycerol, oxalate, citric acid, formic acid and acetic acid, which are commonly found in exudates produced, for instance, by the soil saprotroph *Lyophyllum* sp. strain Karsten into mineral media (propionate as the carbon source). All or some of these compounds presumably constitute chemoattractants for the fungiphiles studied (Nazir et al. 2012; Zhang et al. 2014; Haq et al. 2018). In contrast, *P. caribensis* DSM 13236^T^ was much less able to migrate along fungal hyphae, or utilize fungal-released compounds in spent propionate medium. We thus assume that the aforementioned compounds, in their form in the propionate medium, are less palatable for this organism, but, alternatively, compounds hampering the growth of this organism might have been present. Overall, the results suggest that *P. hospita* DSM 17164^T^ and *P. terrae* DSM 17804^T^ have behavior—upon confrontation with soil fungi—that allows them to be interactive with these, and is thus similar to that of the aforementioned fungiphilic strains. This was clearly different for *P. caribensis* DSM 13236^T^.

### Genomic Analyses Identify a Two-Species Cluster in the Genus *Paraburkholderia*

We surmised that, within the diverse genus *Paraburkholderia*, evolution may have given rise to clusters of species that are both phylogenetically and ecophysiologically similar. Along time, our understanding of bacterial species evolution and relatedness has been based on 1) DNA–DNA hybridization (DDH), 2) phenotype, 3) the sequence of the 16S rRNA gene, 4) MLSA analysis using concatenated housekeeping genes (Estrada-de los Santos et al. 2013), 5) whole-genome sequence analysis (Meier-Kolthoff and Göker 2019), and 6) ANI/TETRA analyses. With respect to the latter, it has been suggested that it may eventually substitute traditional DDH (Richter and Rossello-Mora 2009; Chun et al. 2018; Ciufo et al. 2018). ANI uses pairwise comparisons of shared orthologous protein-encoding genes. It does not require slicing of the genomes into pieces, thus enabling rapid alignment of large sequences (Richter and Rossello-Mora 2009). By substituting the laborious and error-prone DDH, ANI may even accelerate bacterial taxonomy (Goris et al. 2002). Recently, Ciufo et al. (2018) analyzed the levels of agreement (giving “concordant” data) versus disagreement (discordant) among publicly (NCBI) available type strain genomes using a 96% ANI to define species boundaries. Thus, strains identified as *B.* *cepacia* had concordant ANI at 97%, versus discordant ANI (with other species) at 87.63%. We here used comparable ANIm threshold values for our species circumscriptions.

Thus, the ANIm (and TETRA) values found by us confirmed *P. hospita* DSM 17164^T^, *P. terrae* DSM 17804^T^, and *P. caribensis* DSM 13236^T^ to be indeed closely related, with a much tighter linkage between *P. hospita* DSM 17164^T^ and *P. terrae* DSM 17804^T^ on the one hand than between each of these two and *P. caribensis* DSM 13236^T^ on the other hand. *Paraburkholderia* *terrae* DSM 17804^T^ and *P. hospita* DSM 17164^T^ (ANIm/TETRA of 95.42/0.99784) had values at the border of those that delineate species. Here, we posit that the two species constitute one larger species “cluster.” Interestingly, all of the additional four strains derived from soil fungi, that is, BS001, BS110, BS007, and BS437, fell inside this species cluster, and so we surmised fungal interactivity may be one key ecoevolutionary driver of the respective genomes (fig. 3*B*). We propose to coin this species cluster the *P. hospita* species cluster.

Additional arguments for the existence of a species cluster that encompasses the *P. terrae* and *P. hospita* type strains next to strains BS007, BS110, BS437, and BS001, could be found in the great similarities across all of these strains, as evidenced by the phylogenetic analyses and the shared (concatenated) core genes, with a consistent divergence of the cluster from the close relative *P. caribensis* DSM 13236^T^ as well as *P. phymatum* (figs. 1 and 2*A*). Moreover, the species cluster is supported by previous studies that, by other means, also found taxonomic closeness of the two type strains and divergence from both *P. caribensis* DSM 13236^T^ (Nazir et al. 2012; Estrada-de los Santos et al. 2013; Sawana et al. 2014; Beukes et al. 2017) and *P. phymatum* (see figs. 1–3*A*).

There were some other, subtler, differences across the examined strains, for example, the high overlap of COGs between *P. terrae* DSM 17804^T^ and *P. hospita* DSM 17164^T^ (227), in contrast to, for instance, between *P. terrae* DSM 17804^T^ and *P. caribensis* DSM 13236^T^ (83) (fig. 3*C*).

### Arguments for the Renaming of Fungiphilic Strains BS001, BS110, BS007, and BS437 as Members of *Paraburkholderia hospita*

In the foregoing, we provided arguments for the creation of a species cluster named the *P. hospita* species cluster, encompassing *P. hospita* DSM 17164^T^, *P. terrae* DSM 17804^T^, and the four fungiphilic strains examined here, that is, BS001, BS110, BS007, and BS437. Additional evidence suggests this cluster also encompasses the other two *P. hospita* and *P. terrae* strains analyzed. There may be arguments (based on analyses of genome relatedness) that are in favor of mending the two species into just one species. However, we stopped short of redefining the whole cluster as one new species, as more work—based on larger strain/genome numbers—would be required to provide a more solid basis for such a contention. On the basis of the current analyses, strains BS001, BS007, BS110, and BS437 all appeared to be closest related to *P. hospita* DSM 17164^T^ and hence there are strong arguments for the tenet they belong to this species. Here, we propose to rename the fungiphilic strains BS001, BS110, BS007, and BS437 as members of the species *P. hospita*.

### Ecologically Relevant Traits Were Differentially Found among All Strains

#### Lifestyle in Soil

As revealed by the in-depth genomic analyses, *P. hospita* DSM 17164^T^, *P. terrae* DSM 17804^T^, and *P. caribensis* DSM 13236^T^, plus the fungal-derived strains, were all predicted to be ecologically very versatile (fig. 4 and supplementary table 6, Supplementary Material online). The finding in the type strains of genes for all major pathways, in particular the TCA, glycolysis, Entner–Doudoroff, and gluconeogenesis pathways, indicated their metabolic versatility in soil settings. Also, there was consistency in the finding of incomplete/partial pathways across the three type strains. With respect to this finding, it is possible that complementary genes that are potentially key for these pathways were not recognized on the basis of the current data and database entries. Moreover, the finding of diverse siderophore biosynthesis systems was interesting (supplementary table 6, Supplementary Material online), as siderophores—as iron capturing systems—are important in most soils, particularly in situations in which iron is limiting. They have been observed in other soil-derived *Paraburkholderia* strains, for example, *Paraburkholderia* *xenovorans* (Vargas-Straube et al. 2016), strain BS001 and even in the horizontal gene pool in mycospheres (Zhang et al. 2016).

The analyses further showed the genomes of *P. terrae* DSM 17804^T^ and *P. hospita* DSM 17164^T^ to contain sets of genes potentially involved in fungal-interactive next to saprotrophic behaviors. This indicated that these organisms, notwithstanding their fungal interactivity potential, constitute ecological “generalists” rather than specialists (Haq et al. 2014). Fungal-interactive capacities presumably involve several metabolic response genes (e.g., the five-gene cluster), next to genes for motility/chemotaxis, T3SS, T4P, and biofilm formation, much like found earlier in the fungal-interactive strain BS001 (Warmink and van Elsas 2009; Haq et al. 2014; Yang et al. 2016, 2017). Furthermore, the genomic analyses revealed an enormous capacity in all strains for both primary and secondary metabolisms (supplementary tables 6 and 7, Supplementary Material online). On the basis of these capabilities, the lifestyles of these strains in soil may be depicted as multi-faceted, including saprotrophic next to host-interactive phases. With respect to saprotrophy, the presence of a variety of carbohydrate metabolism genes indicated a clear capacity of the *P. hospita* species cluster strains to be involved in “spurs” of degradation of the respective polymer substrates (see fig. 6). In detail, the presence of genes related to chitin degradation (CH19 and CH75) was suggestive of fungal-interactive behavior of members of this species cluster, as fungal cell walls contain chitins, next to chitosan and glucans (Zhao et al. 2013; Shinya et al. 2016; Kusaoke et al. 2017). The finding of a gene encoding a GH5 enzyme uniquely in *P. terrae* DSM 17804^T^ was surprising; this gene endows its host with endo-β-1,4-mannanase (EC: 3.2.1.78) or plant/fungal cell-wall-degrading enzymes, and so backbones of mannan polysaccharides may be transformed into oligosaccharides (Latgé 2007; Engel et al. 2012; Busch et al. 2017).

#### Secretion Systems and Putative Roles

The finding of copies of the T1SS, T2SS, T3SS, and T6SS across the three type strains as well as the further strains of the same species (next to the four fungiphiles) points to the importance of such systems in the contact of soil-dwelling cells with their surroundings, including living cells (fig. 4*A* and supplementary fig. 3, Supplementary Material online). Of all of these systems, the T3SS has been indicated to confer an enhanced capacity to the host to adhere to fungal surfaces, endowing cells with an adherence/injection device (Warmink and van Elsas 2008; Haq et al. 2014; Yang et al. 2016, 2017). The finding of ample T3SSs across these organisms contrasted with the notion that many *Paraburkholderia* species lack such systems (Estrada-de los Santos et al. 2016). Moreover, the finding of differential presence/absence of the T4SS across *P. hospita* DSM 17164^T^, *P. terrae* DSM 17804^T^ (presence in both), and *P. caribensis* DSM 13236^T^ (absence) points at a differential legacy of interactive (i.e., horizontal gene transfer—HGT) events across the former two organisms versus the latter one.

#### Glycerol and Oxalate Transformations and Biofilms

Membrane transporters are tightly related to the nutrient and mineral fluxes that are necessary to maintain the biological processes in living cells. The glycerol and oxalic acid released by *Lyophyllum* sp. strain Karsten can serve as carbon sources as well as molecular signals for *Paraburkholderia*, much like shown for strain BS001 (Haq et al. 2016, 2017). Here, we addressed the potential of *P. hospita* DSM 17164^T^, *P. terrae* DSM 17804^T^, and *P. caribensis* DSM 13236^T^ to capture and metabolize these compounds. Clearly, the OFA family transporters found might be important in a biochemical cycle consisting of: 1) oxalate influx, 2) oxalate decarboxylation, and 3) formate efflux. This energy generating system has been intensively studied in *Oxalobacter formigenes* (Anantharam et al. 1989).

Moreover, the five-gene cluster present across all *Paraburkholderia* strains studied here was previously reported to be significantly expressed in (strain BS001) cells that are in contact with fungal hyphae (Haq et al. 2017), as an energy generating system. Its conservation in *P. hospita* DSM 17164^T^ (and other *P hospita* strains), *P. terrae* DSM 17804^T^ (and other *P. terrae* strains), and the four fungiphiles may indicate its importance in the strains of the two-species cluster, in processes similar to those in selected fungal-derived *Paraburkholderia* strains (fig. 7).

In the light of the importance of biofilm formation by *Paraburkholderia* at fungal surfaces (Warmink et al. 2011; Haq et al. 2016), we analyzed the genomes of *P. hospita* DSM 17164^T^, *P. terrae* DSM 17804^T^, and *P. caribensis* DSM 13236^T^, and other strains of these three species, for the presence of biofilm formation genes (fig. 4 and supplementary fig. 3, Supplementary Material online). The fact that the *pel*, *pga* and alginate-related genetic systems were found across the three genomes indicates their importance in all three type strains. Here, the analyses of synteny showed high similarity within the *P. terrae*/*P. hospita* genomes versus lower similarity of this group with the *P. caribensis* genome. Hence, eco-evolutionary divergent paths were found across these two groups, again testifying that their lines of descent are different.

#### Mobile genetic elements

The lifestyles of the investigated *Paraburkholderia* strains in soil may be strongly determined by the RGPs they harbor, and hence HGT may be a main facet shaping both the fitness of the organisms and their genomes. Indeed, we found clear evidence for the presence of T4SS-like mobility systems that were linked to GIs in *P. terrae* DSM 17804^T^ (RGP97) and *P. hospita* DSM 17164^T^ (RGP99) (see supplementary tables 9 and 10, Supplementary Material online), versus the absence of such inserts from *P. caribensis*. The huge genome size of *P. hospita* DSM 17164^T^ (11.5 Mb), next to its easy capture of the pEMT1 plasmid in soil, may indicate that 1) *P. hospita* DSM 17164^T^ is an avid recipient of exogenous genetic material, enabling it to rapidly adjust its genomic complement to the vagaries of the environment (Dagan et al. 2008), and 2) *P. hospita* DSM 17164^T^ has been subjected to frequent events of HGT and, presumably, positive selection of foreign DNA. Consistent with this tenet was the finding of high numbers of transposases and integrases in the genome of this organism. It is possible that *P. terrae* DSM 17804^T^ (and *P. caribensis* DSM 13236^T^) has the same capabilities, but has been subjected to a lower number of HGT and selection events. However, the extent to which different types of MGEs have entered the genomes of the three type strains, are maintained or deleted there, and endow the host with fitness traits still needs further analysis (Pratama and van Elsas 2019).

### Overall Conclusions

The genomes of *P. terrae* DSM 17804^T^, *P. hospita* DSM 17164^T^ and *P. caribensis* DSM 13236^T^ (9–11.5 Mb) are, as previously reported for, for example, fungiphilic strain BS001, among the largest bacterial genomes described. Clearly, these genome sizes fall within the range of sizes previously reported for the four fungiphilic strains (selectively included in the analyses presented here). The evaluation of the ANIm (and TETRA) values across our strain set revealed the existence of a tight species cluster typified by *P. hospita* DSM 17164^T^ (and named the *P. hospita* species cluster), which includes *P. hospita* DSM 17164^T^, *P. terrae* DSM 17804^T^ and the four fungiphilic strains BS001, BS007, BS110, and BS437, but excludes *P. caribensis* DSM 13236^T^. Further supporting evidence for this relatedness was found in the phylogenetic analyses (extended with additional genomes) as well as the shared orthologous genes between these strains.

Based on the collective data, we propose to: 1) reassign the formerly named *P. terrae* strains BS001, BS007, BS110, and BS437 to *P. hospita* and 2) group the *P. terrae/P. hospita*-like strains within one species cluster named the *P. hospita* species cluster. To reach this conclusion, we took into account our own ANIm and TETRA analyses, the phenotypic analyses, DDH results reported previously (Goris et al. 2002; Yang et al. 2006) and the ANI cutoff values for *Burkholderia* species (Ciufo et al. 2018). Genomic analyses (comparative and CAZyme) of the two type strains, next to the four fungiphiles, revealed these all share traits conferring interactivity with soil organisms such as fungi, which is consistent with a presumed common ancestry and ecophysiology. The traits that were used as “reporters” of fungal interactivity capacities included secretion systems, type-4 pili, biofilm formation, flagellar motility and chemotaxis, the five-gene metabolic response cluster and glycerol and oxalate uptake systems. By the criteria applied, *P. caribensis* was not strongly fungal-interactive and it was also genomically divergent. Overall, our results confirm the tight relatedness of *P. hospita* DSM 17164^T^, *P. terrae* DSM 17804^T^ and the fungiphiles BS001, BS007, BS110, and BS437. We extended our analyses to other currently available genomes of the three species, and suggest that this analysis indeed indicates that a cluster of soil organisms with fungal-associative behavior exists around the species *P. hospita* and *P. terrae*.

## Supplementary Material

evaa031_Supplementary_Data
