# Supplementary material for: Delineation of a Subgroup of the Genus Paraburkholderia, Including P. terrae DSM 17804T, P. hospita DSM 17164T, and Four Soil-Isolated Fungiphiles, Reveals Remarkable Genomic and Ecological Features—Proposal for the Definition of a P. hospita Species Cluster
Source: Genome Biol Evol. 2020 Feb 18;12(4):325–44. doi: 10.1093/gbe/evaa031 (PMC7186790; doi:10.1093/gbe/evaa031)
Supplement: evaa031_Supplementary_Data [file evaa031_supplementary_data.zip › GBE-typestrains-Supmaterial-rev2final.docx]

**Supplementary Figures**

**Delineation of a subgroup of the genus *Paraburkholderia,* including *P. terrae* DSM 17804^T^, *P. hospita* DSM 17164^T^ and four soil-isolated fungiphiles, reveals remarkable genomic and ecological features – Proposal for the definition of a *P. hospita* ’species cluster’**

Akbar Adjie Pratama^1^, Diego Javier Jiménez^2^, Qian Chen^1^, Boyke Bunk^3^, Cathrin Spröer^3^, Jörg Overmann^3,4^ and Jan Dirk van Elsas^1^

^1^Department of Microbial Ecology - Groningen Institute for Evolutionary Life Sciences, University of Groningen, Nijenborgh 7, Groningen, 9747 AG, The Netherlands.

^2^Microbiomes and Bioenergy Research Group, Department of Biological Sciences, Universidad de los Andes, Carrera 1 No 18A-12, Bogotá, Colombia.

^3^Leibniz Institute DSMZ-German Collection of Microorganisms and Cell Cultures, Inhoffenstraße 7 B, 38124 Braunschweig, Germany

^4^Microbiology, Braunschweig University of Technology, Braunschweig, Germany

Corresponding author: JDvE


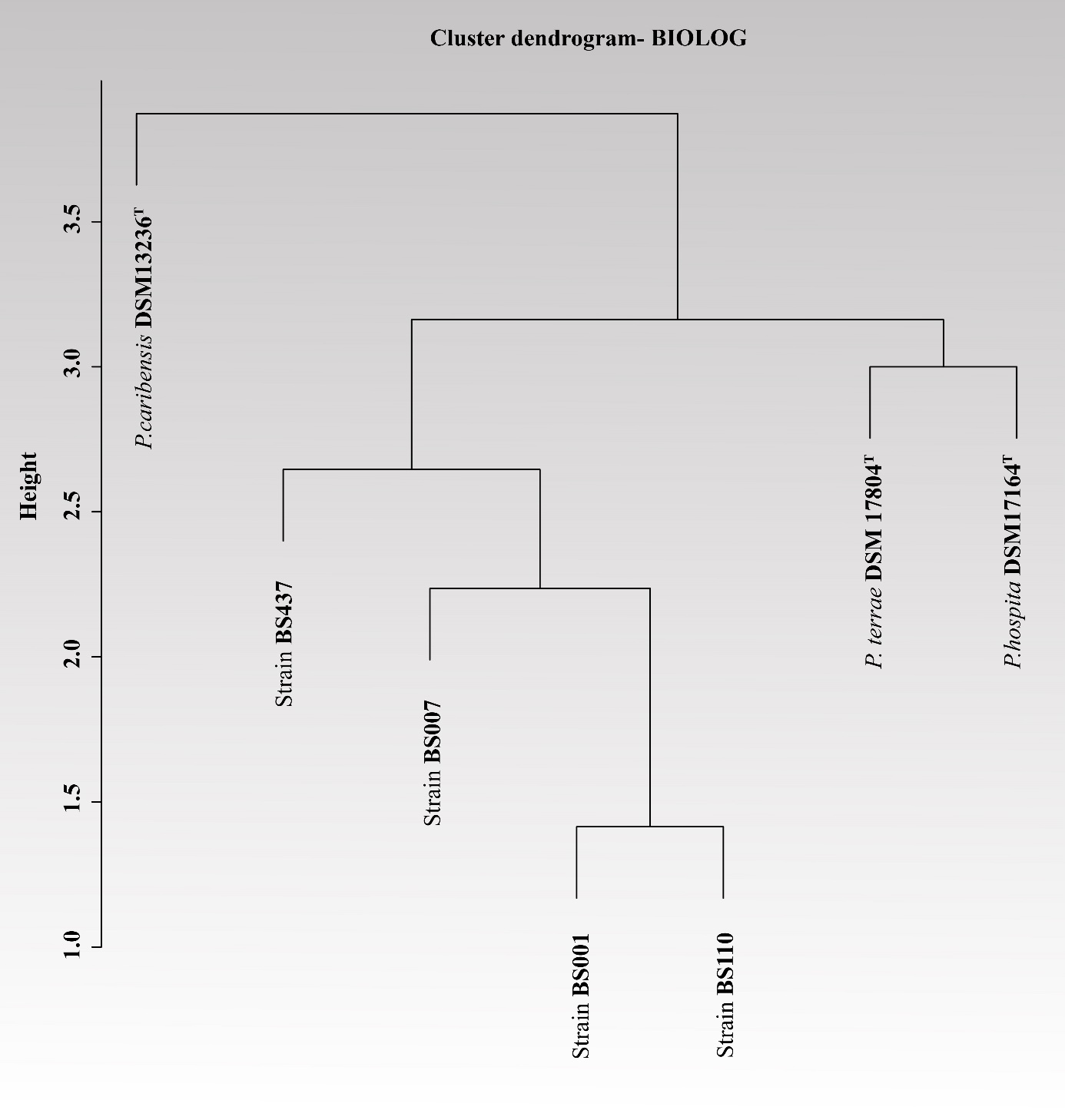


**Supplementary Figure 1**. Hierarchical cluster analysis of carbon utilization patterns observed in BIOLOG plates for *Paraburkholderia terrae* DSM 17804^T^, *P.hospita* DSM 17164^T^, *P.caribensis* DSM 13236^T^, and fungiphilic strains BS001, BS007, BS110 and BS437.


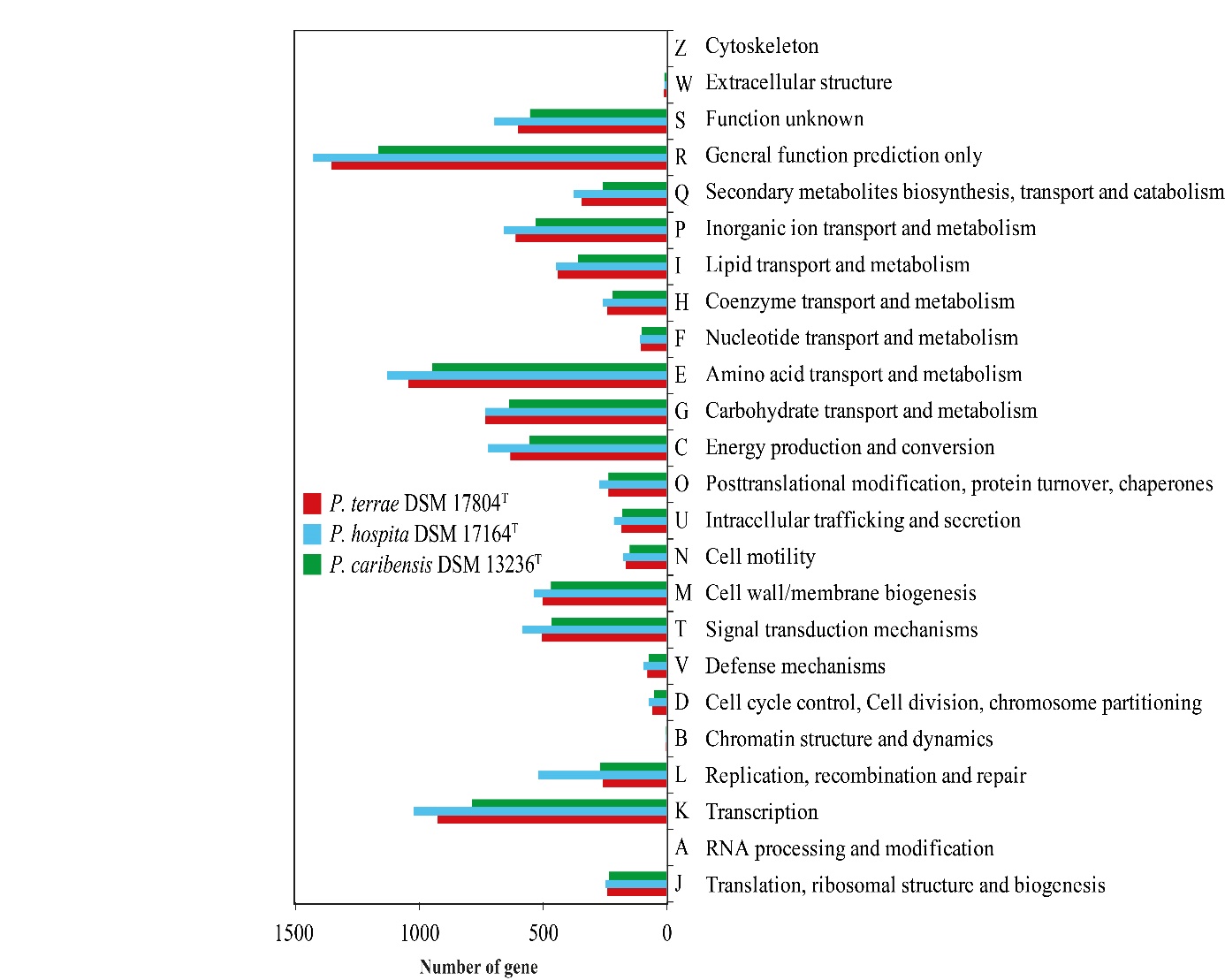


**Supplementary Figure 2**. Number of genes associated with general COG functional categories of *P. terrae* DSM 17804T, *P. hospita* DSM 17164^T^ and *P.caribensis* DSM 13236^T^ (as reported by Microscope platform).


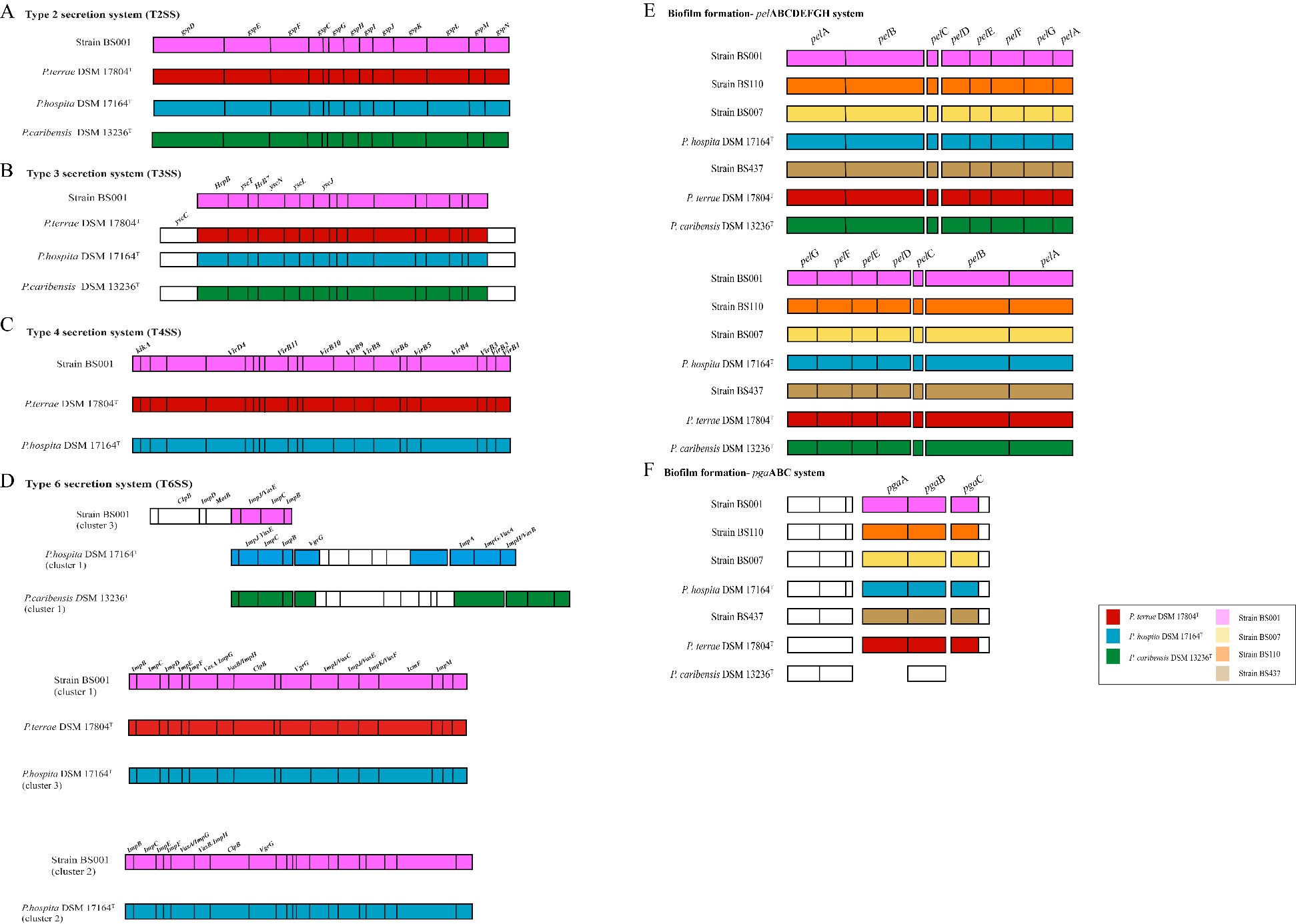


**Supplementary Figure 3**. Synteny reconstruction of (**A**) T2SS, (**B**) T3SS, (**C**) T4SS, (**D**) T6SS and biofilm formation genes, i.e. (**E**) pel and (**F**) pga systems across *Paraburkholderia terrae* DSM 17804^T^, *P. hospita* DSM 17164^T^ and *P. caribensis* DSM 13236^T^, next to BS001, BS007, BS110 and BS437. Further synteny analyses can be seen in: secretion systems of *P. terrae* BS001 (Haq, Graupner, Nazir, & Van Elsas, 2014), BS007, BS110 and BS437 (Pratama, Haq, Nazir, Chaib De Mares, & van Elsas, 2017). Color codes based on the genomes as indicated.


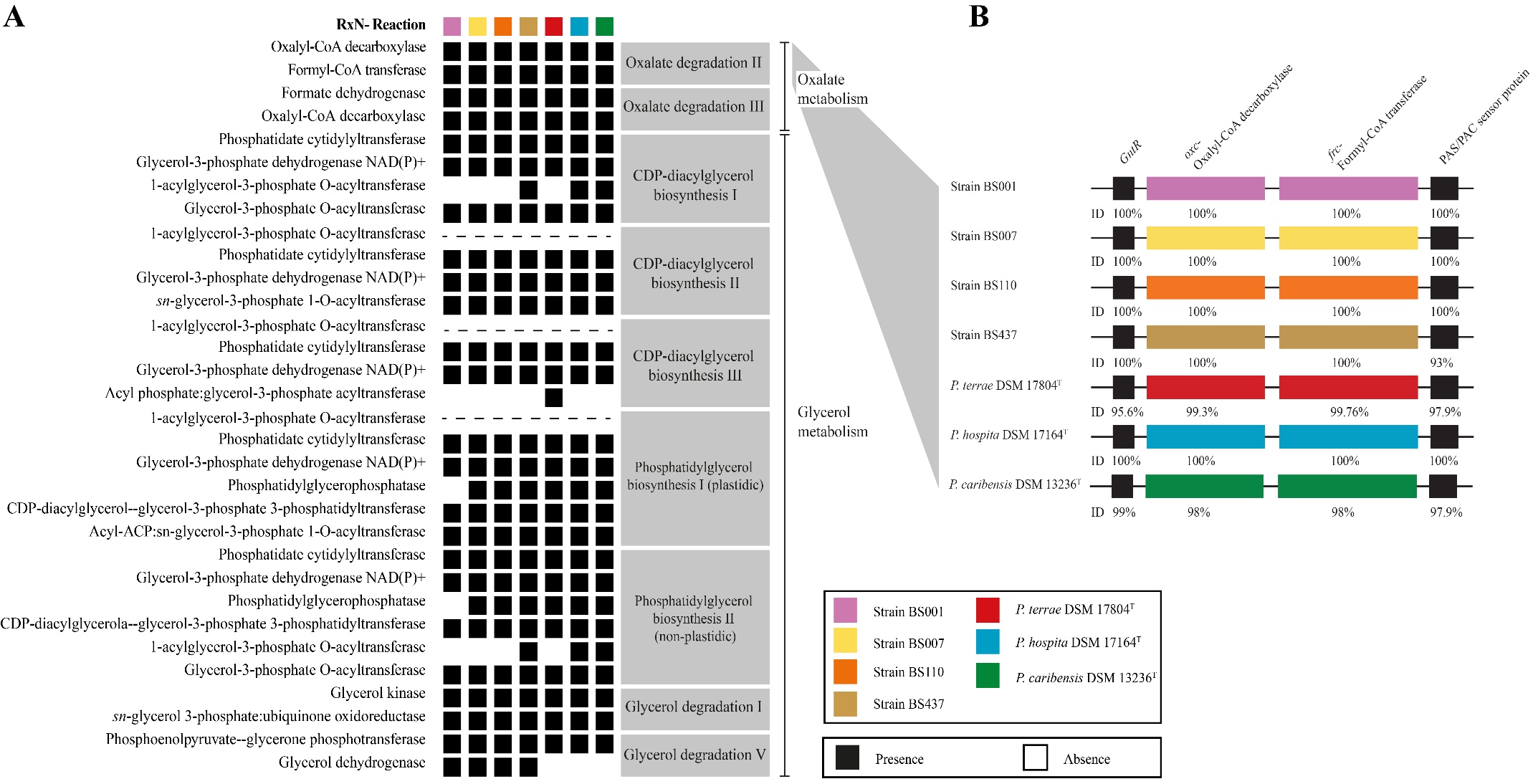


**Supplementary Figure 4**. (**A**) The absence and presence of oxalate and glycerol metabolisms found in all type strains, and strains BS001, BS007, BS110 and BS437. Percentage similarity of genes for *oxc* – oxalyl-CoA decarboxylase and *frc*-formyl-CoA transferase across the genomes. (**B**) Synteny analyses of *oxc* – oxalyl-CoA decarboxylase and *frc*-formyl-CoA transferase across genomes. Color codes based on the genomes as indicated.


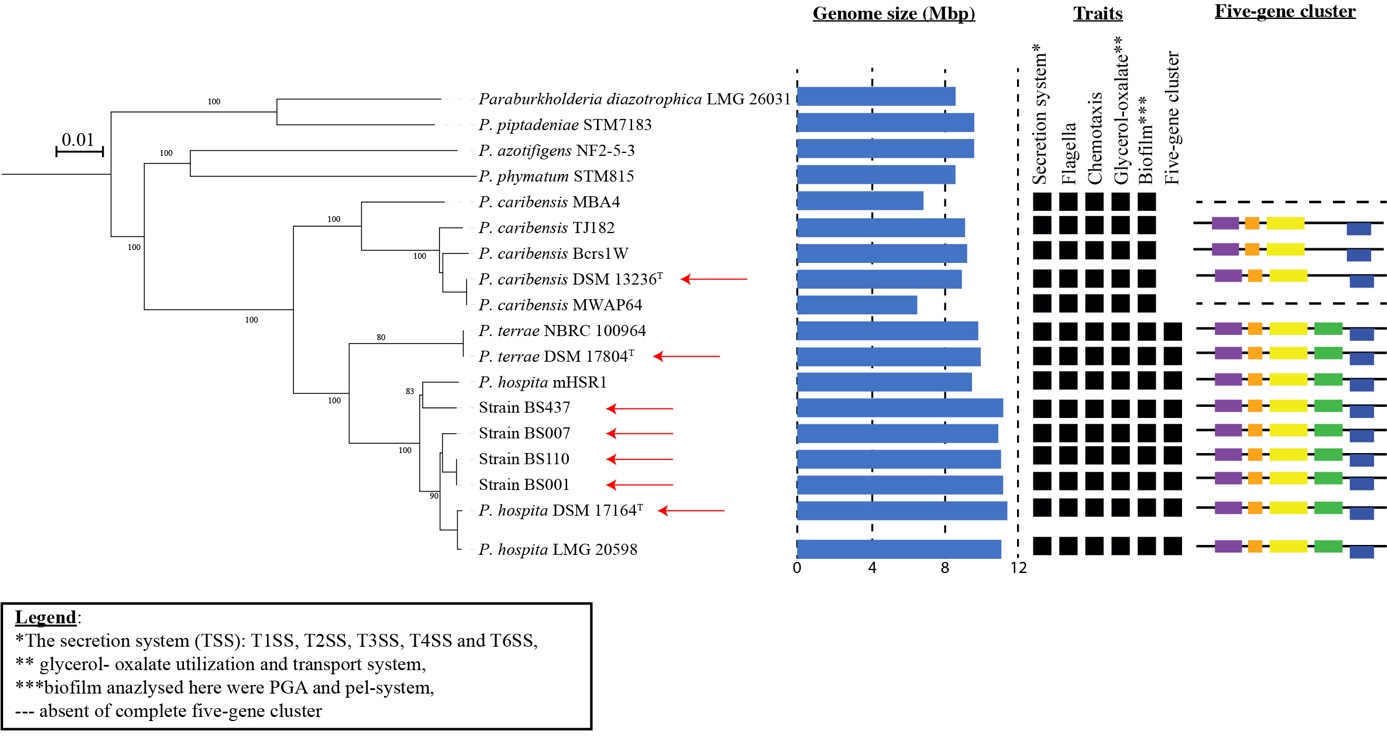


**Supplementary Figure 5**. Comparison of the genomes of *Paraburkholderia terrae* DSM 17804^T^, *P. hospita* DSM 17164^T^, *P. caribensis* DSM 13236^T^ and other strains available in the public database. Here we place emphasis on the clustering and genome sizes and selected traits that are potentially important for bacterium-fungus interactions, i.e. genes for diverse secretion systems, flagella, chemotaxis, glycerol-oxalate uptake and utilization, biofilm formation and (five-gene) metabolic/detoxification clusters (present/absent matrices). The phylogenetic tree was built using whole-genome sequence analysis in the type (strain) genome server – TYGS (https://tygs.dsmz.de/). Bootstrap confidence values ≥ 70% are indicated.
